# Supplementary figures and images for: Mouse-Specific Single cell cytokine activity prediction and Estimation (MouSSE)
Source: PLoS Comput Biol. 2025 Sep 19;21(9):e1013475. doi: 10.1371/journal.pcbi.1013475 (PMC12469201; doi:10.1371/journal.pcbi.1013475)

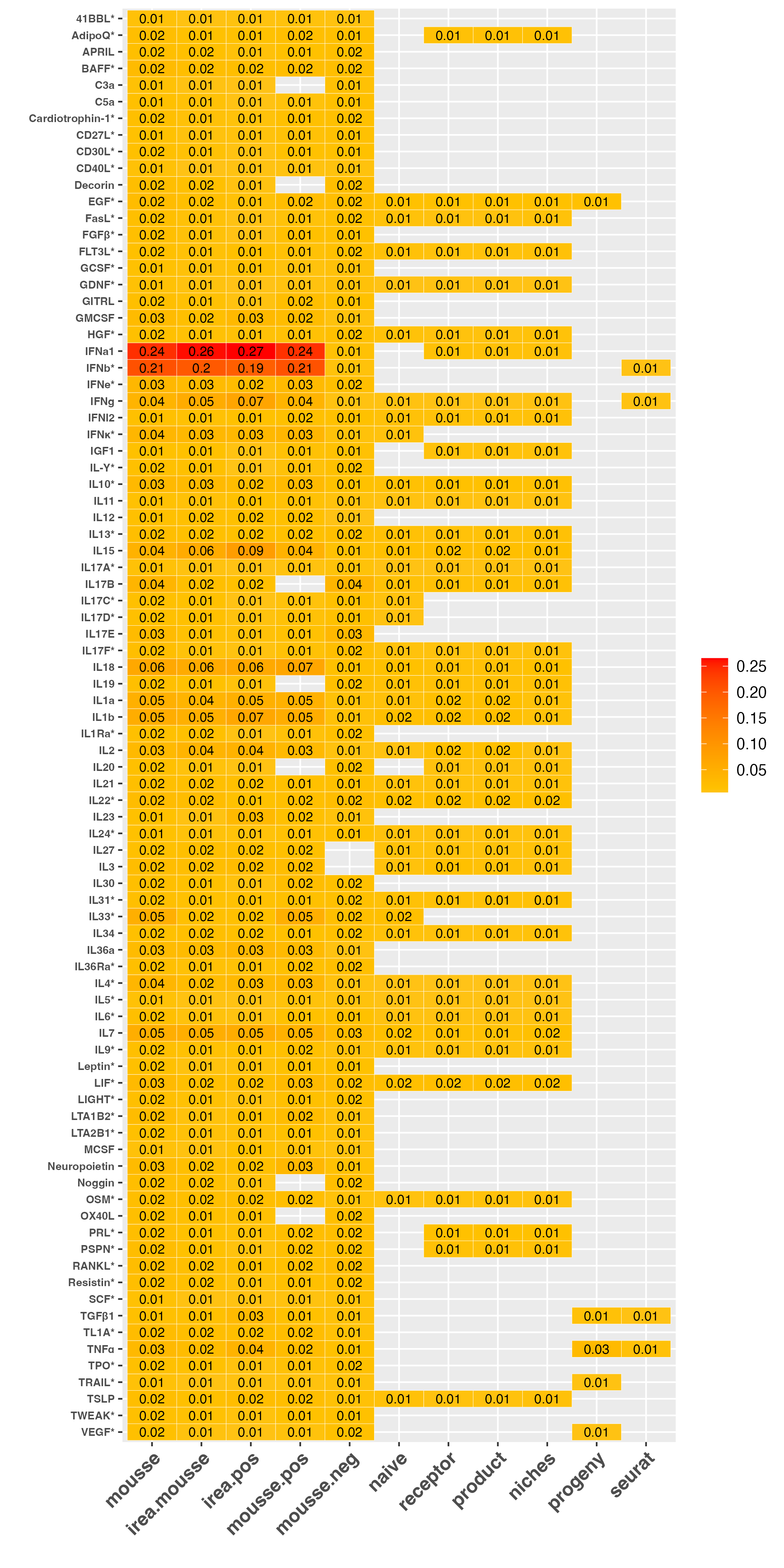

Supplement: S1 Fig — Cytokine markers with an asterisk have the highest PR-AUC score when estimated using the MouSSE method (mousse). (TIFF) [file pcbi.1013475.s001.tiff]

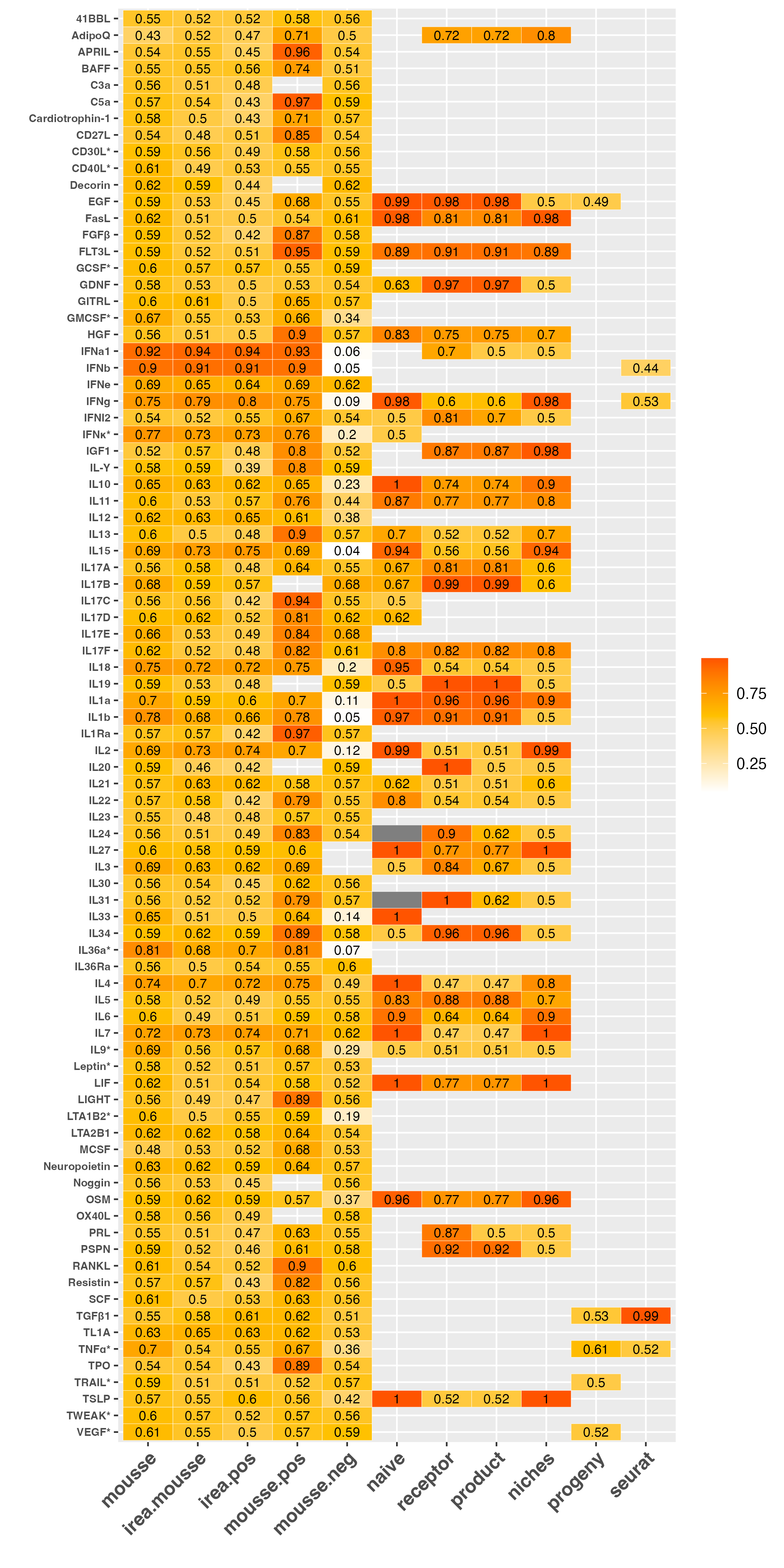

Supplement: S2 Fig — Cytokine markers with an asterisk have the highest specificity when estimated using the MouSSE method (mousse). (TIFF) [file pcbi.1013475.s002.tiff]

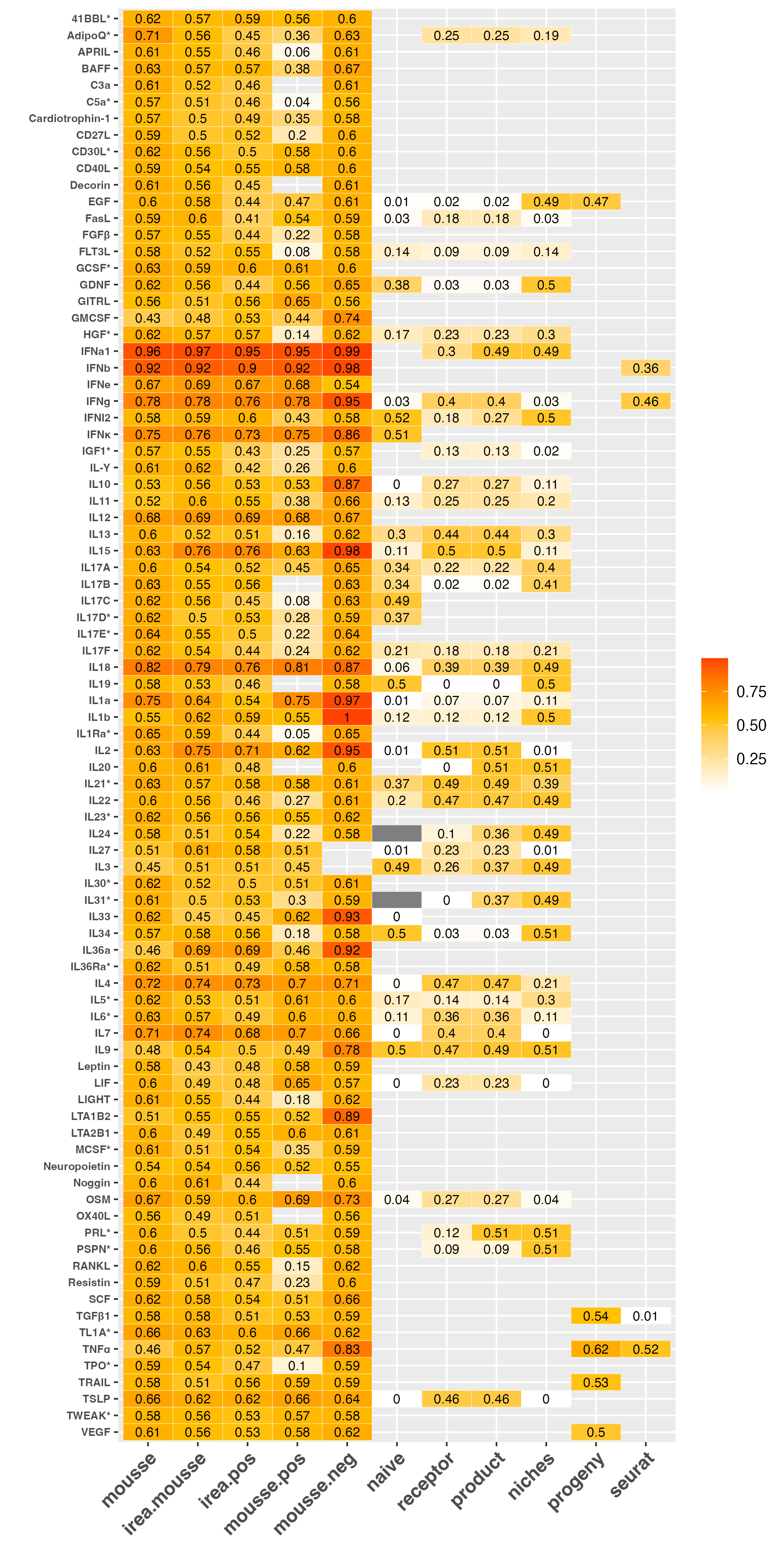

Supplement: S3 Fig — Cytokine markers with an asterisk have the highest sensitivity when estimated using the MouSSE method (mousse). (TIFF) [file pcbi.1013475.s003.tiff]

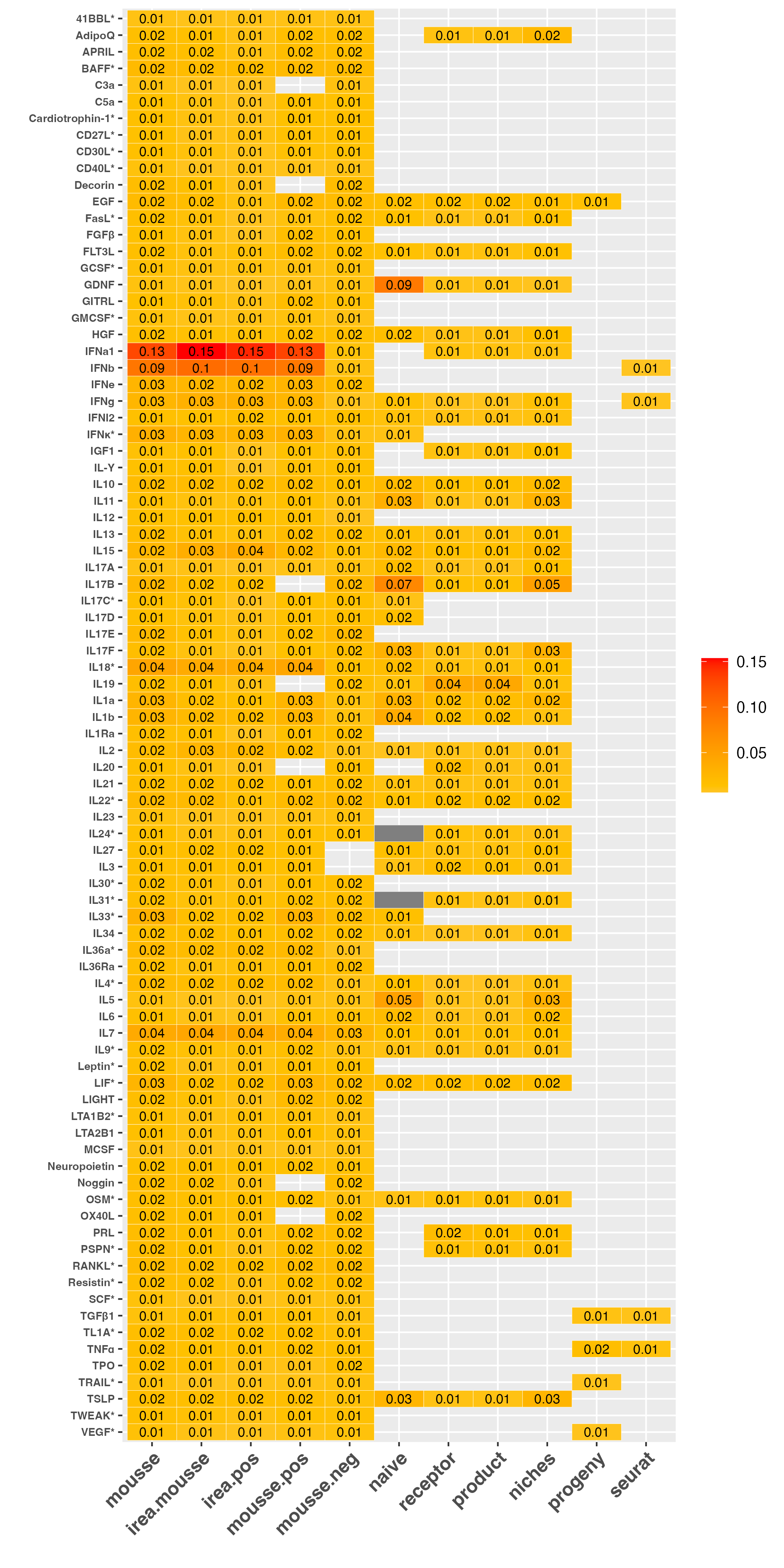

Supplement: S4 Fig — Cytokine markers with an asterisk have the highest precision when estimated using the MouSSE method (mousse). (TIFF) [file pcbi.1013475.s004.tiff]

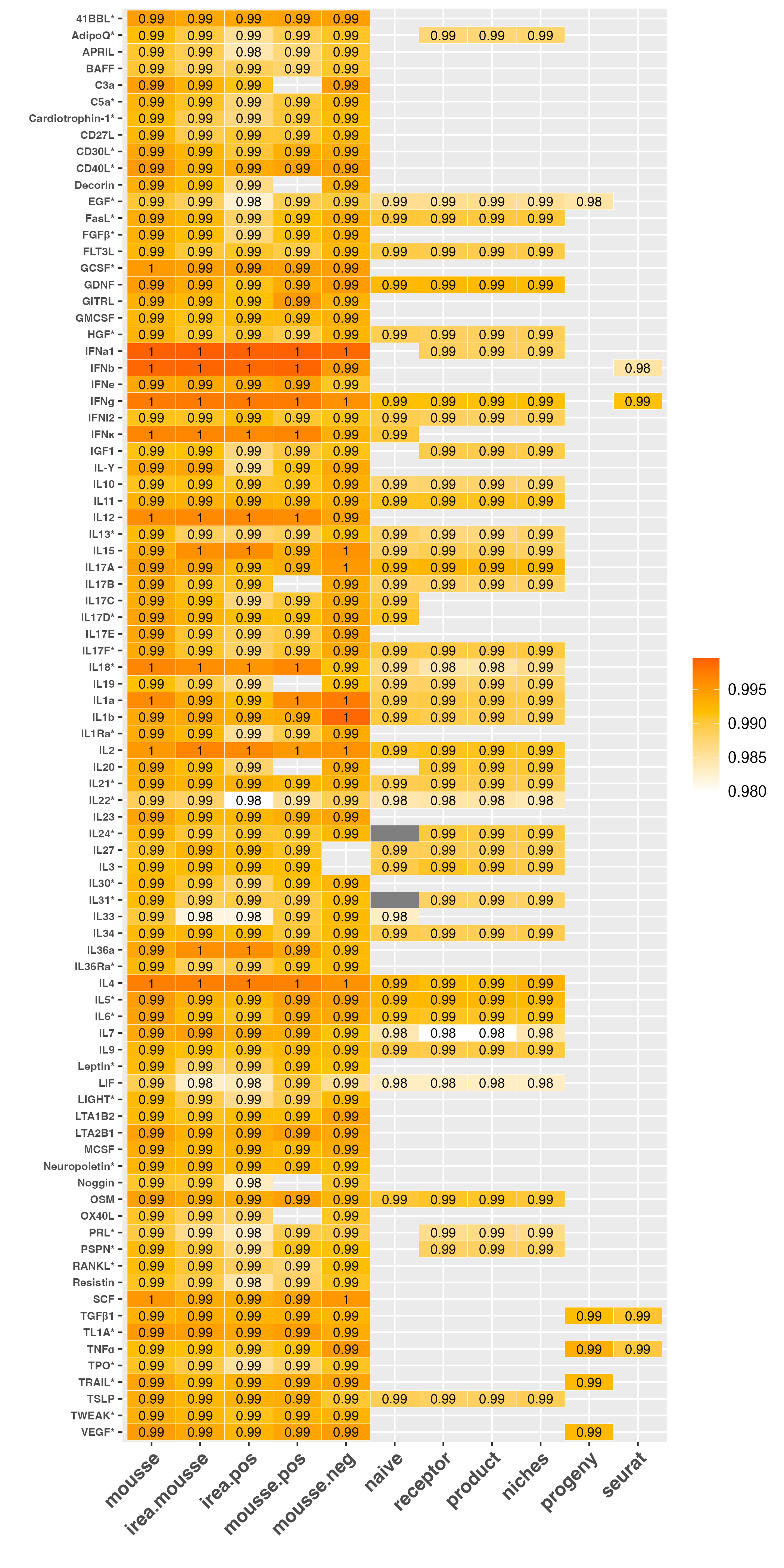

Supplement: S5 Fig — Cytokine markers with an asterisk have the highest NPV when estimated using the MouSSE method (mousse). (TIFF) [file pcbi.1013475.s005.tiff]

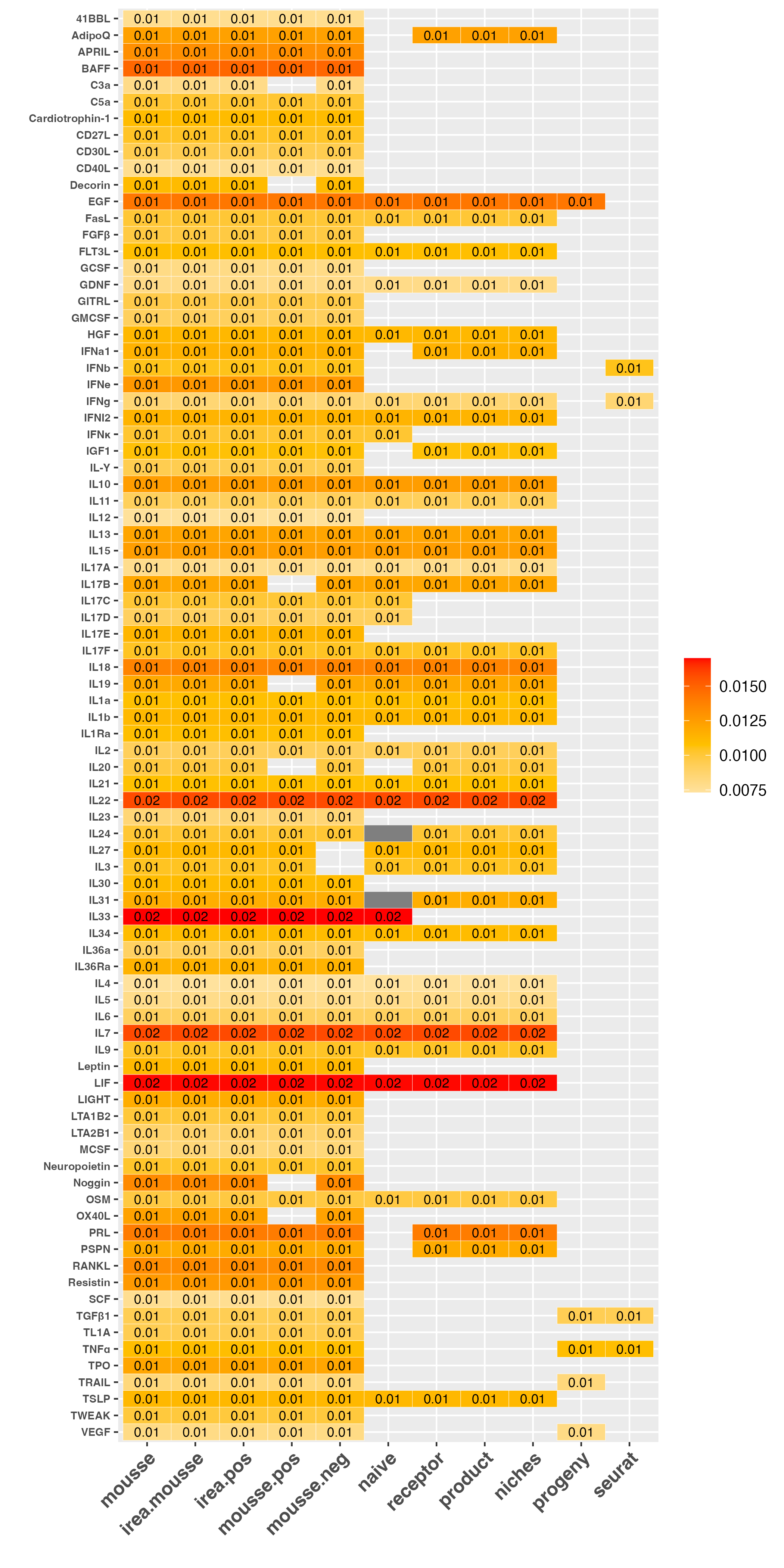

Supplement: S6 Fig — Cytokine markers with an asterisk have the highest prevalence when estimated using the MouSSE method (mousse). (TIFF) [file pcbi.1013475.s006.tiff]

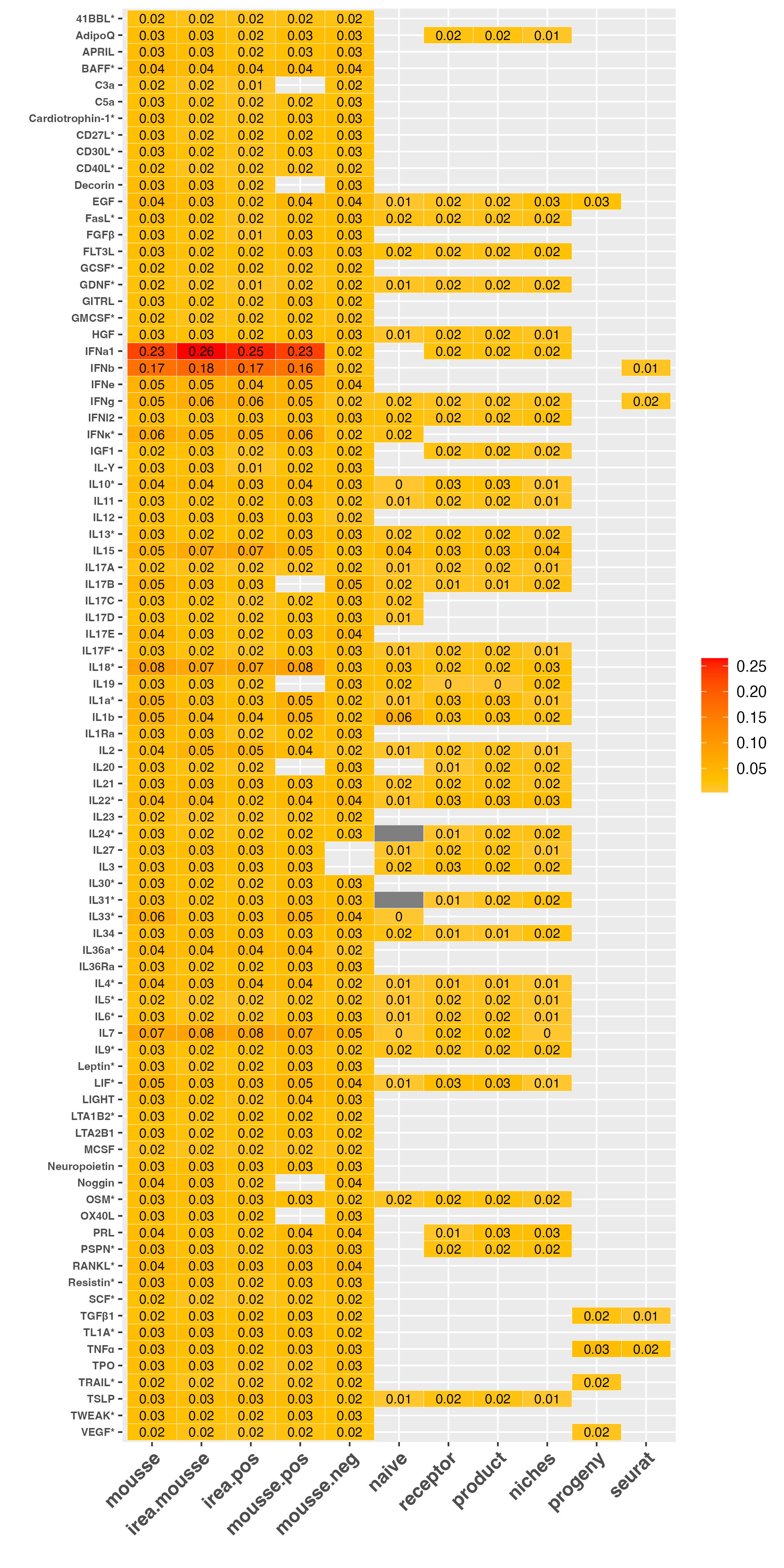

Supplement: S7 Fig — Cytokine markers with an asterisk have the highest F1 score when estimated using the MouSSE method (mousse). (TIFF) [file pcbi.1013475.s007.tiff]

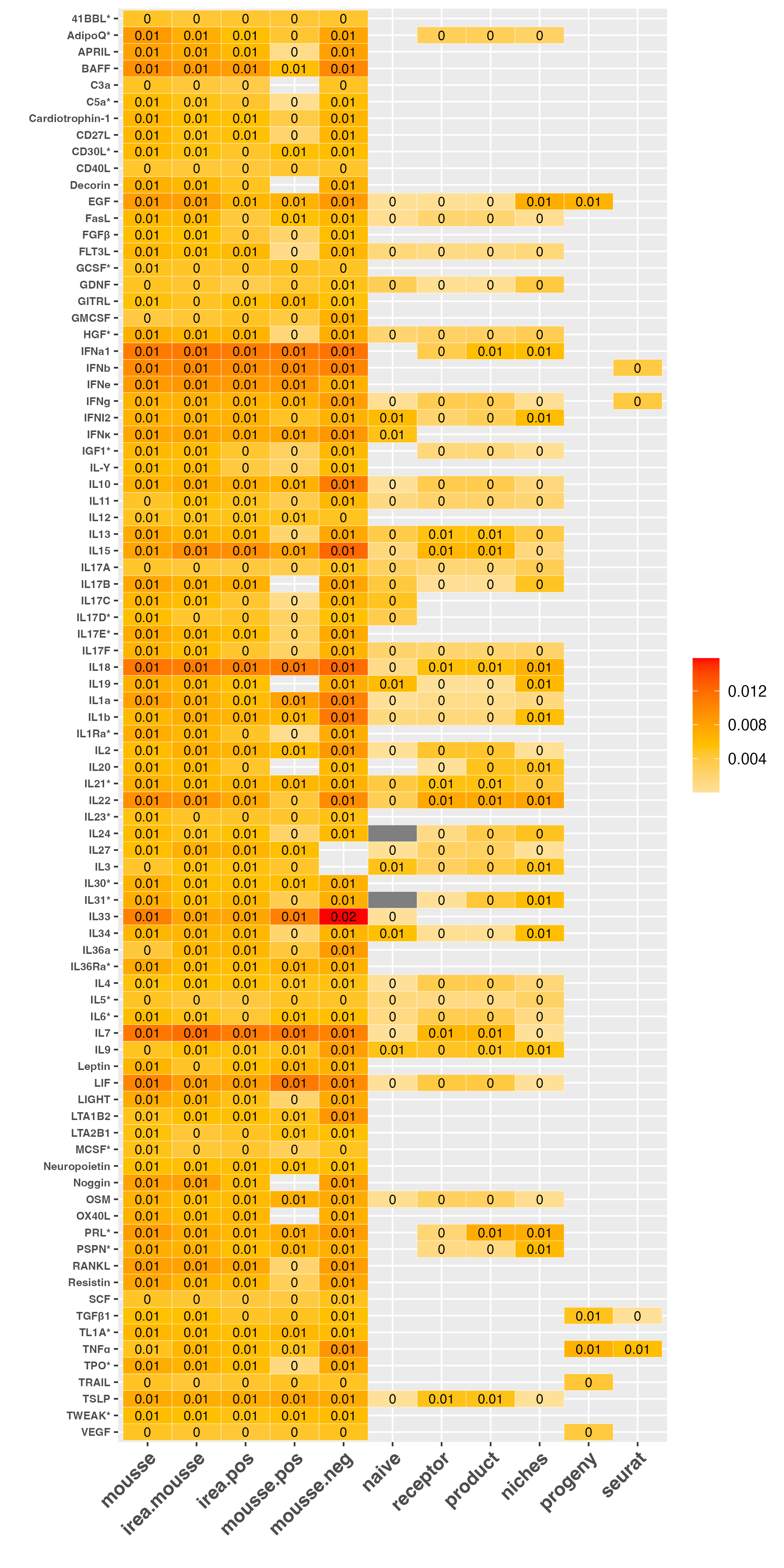

Supplement: S8 Fig — Cytokine markers with an asterisk have the highest detection rate when estimated using the MouSSE method (mousse). (TIFF) [file pcbi.1013475.s008.tiff]

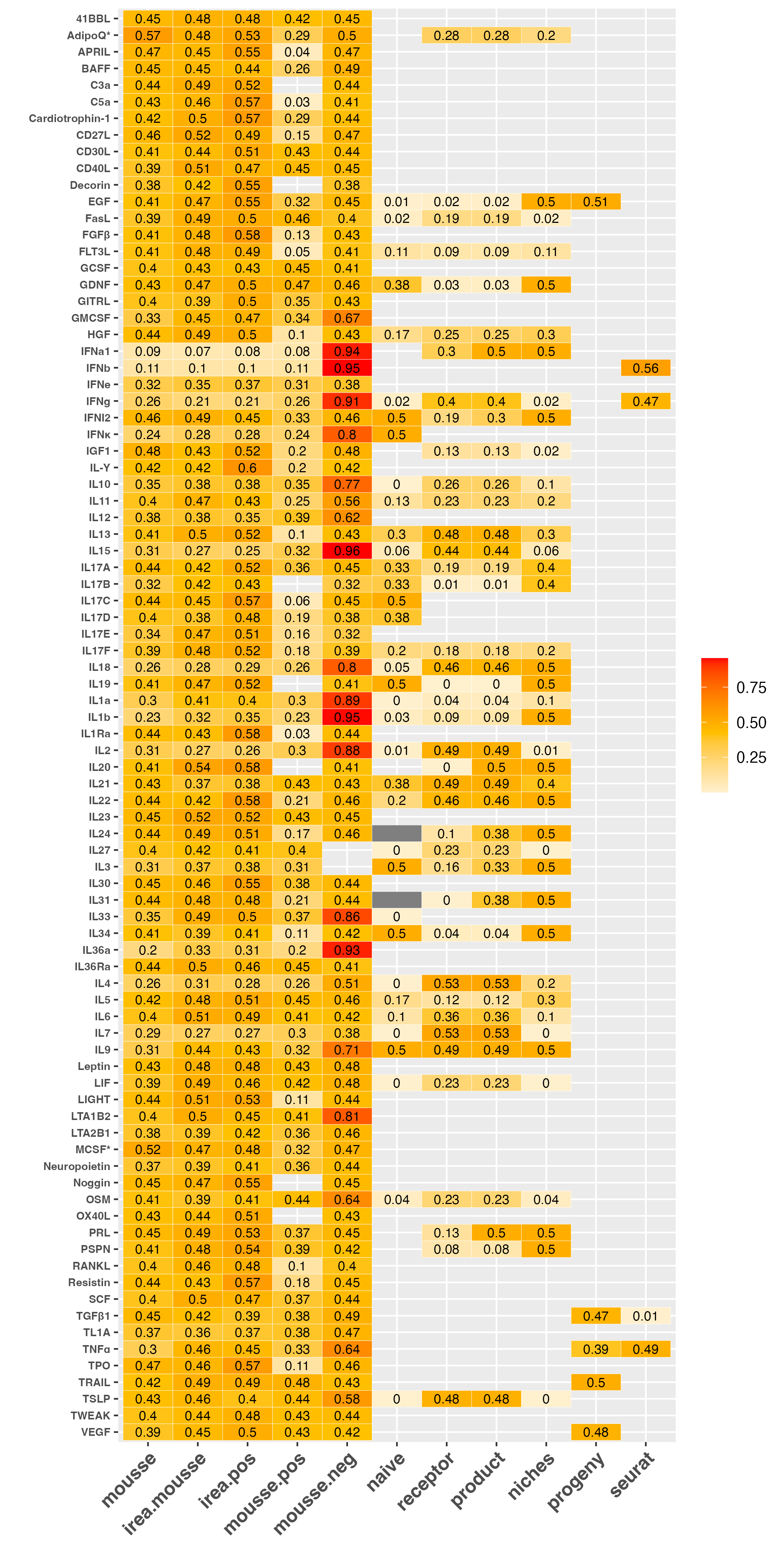

Supplement: S9 Fig — Cytokine markers with an asterisk have the highest detection prevalence when estimated using the MouSSE method (mousse). (TIFF) [file pcbi.1013475.s009.tiff]

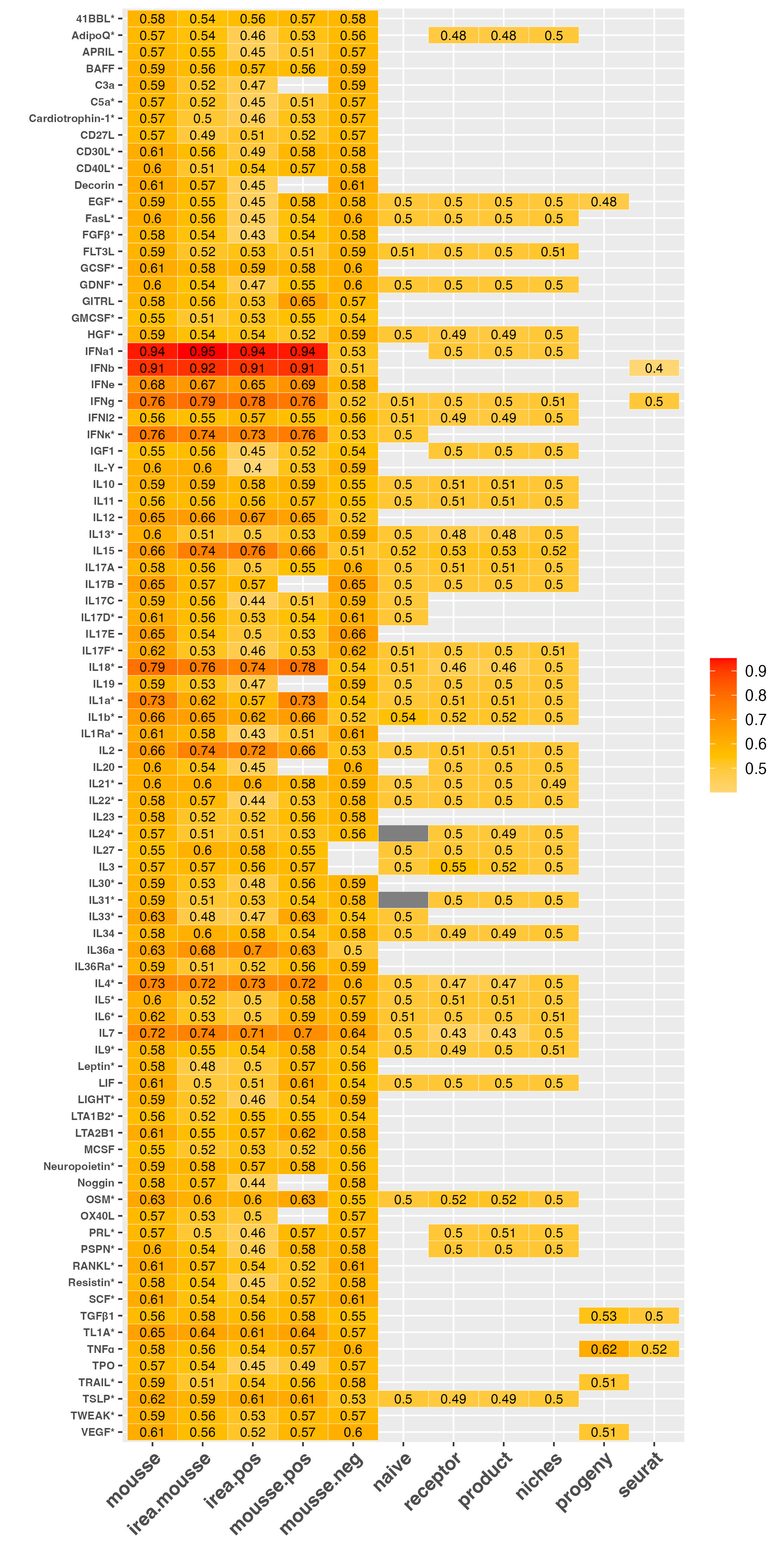

Supplement: S10 Fig — Cytokine markers with an asterisk have the highest balanced accuracy when estimated using the MouSSE method (mousse). (TIFF) [file pcbi.1013475.s010.tiff]

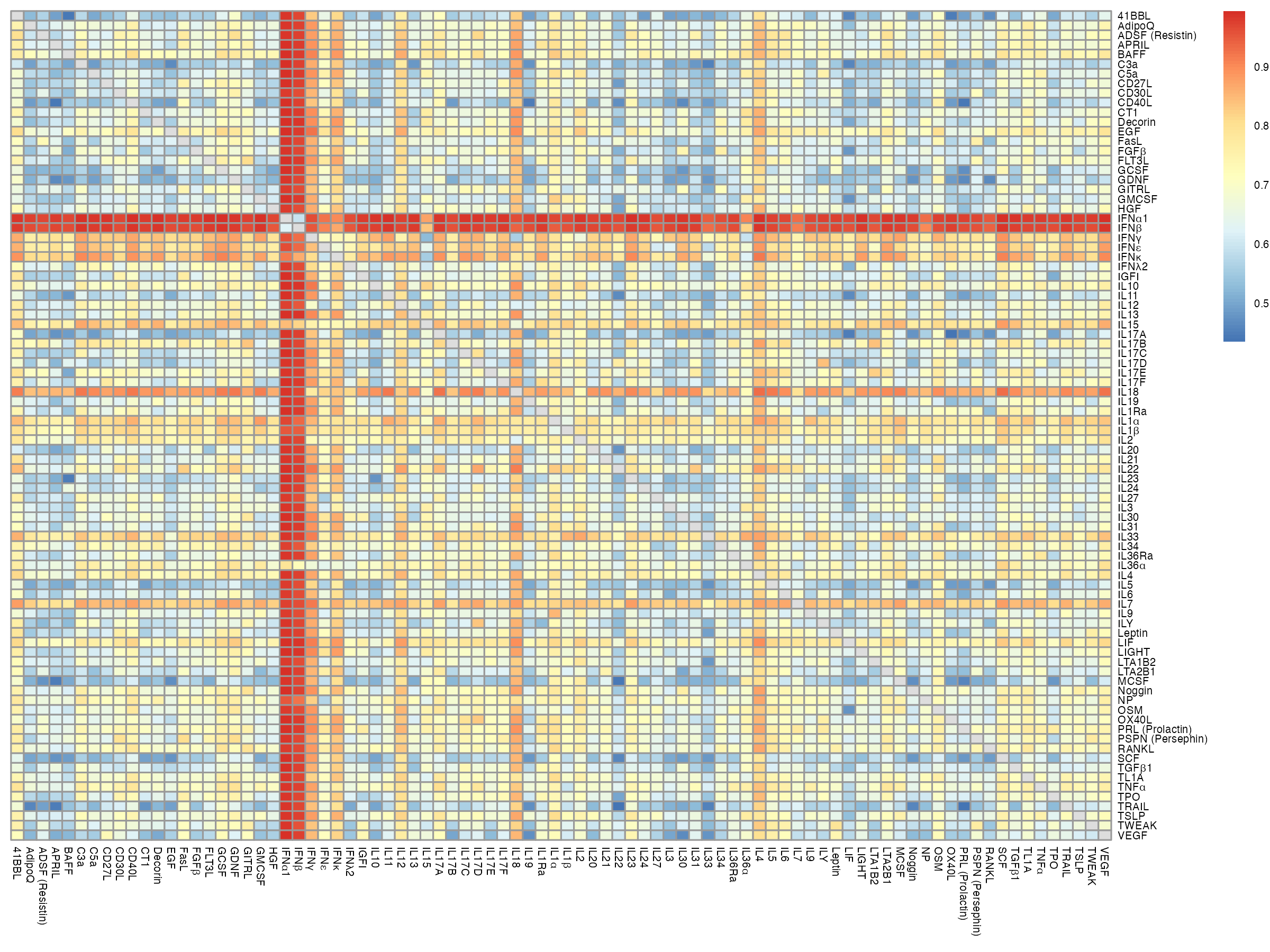

Supplement: S11 Fig — Rows represent cytokines to defined markers for (i.e., cytokines that are differentiated for). (TIFF) [file pcbi.1013475.s011.tiff]

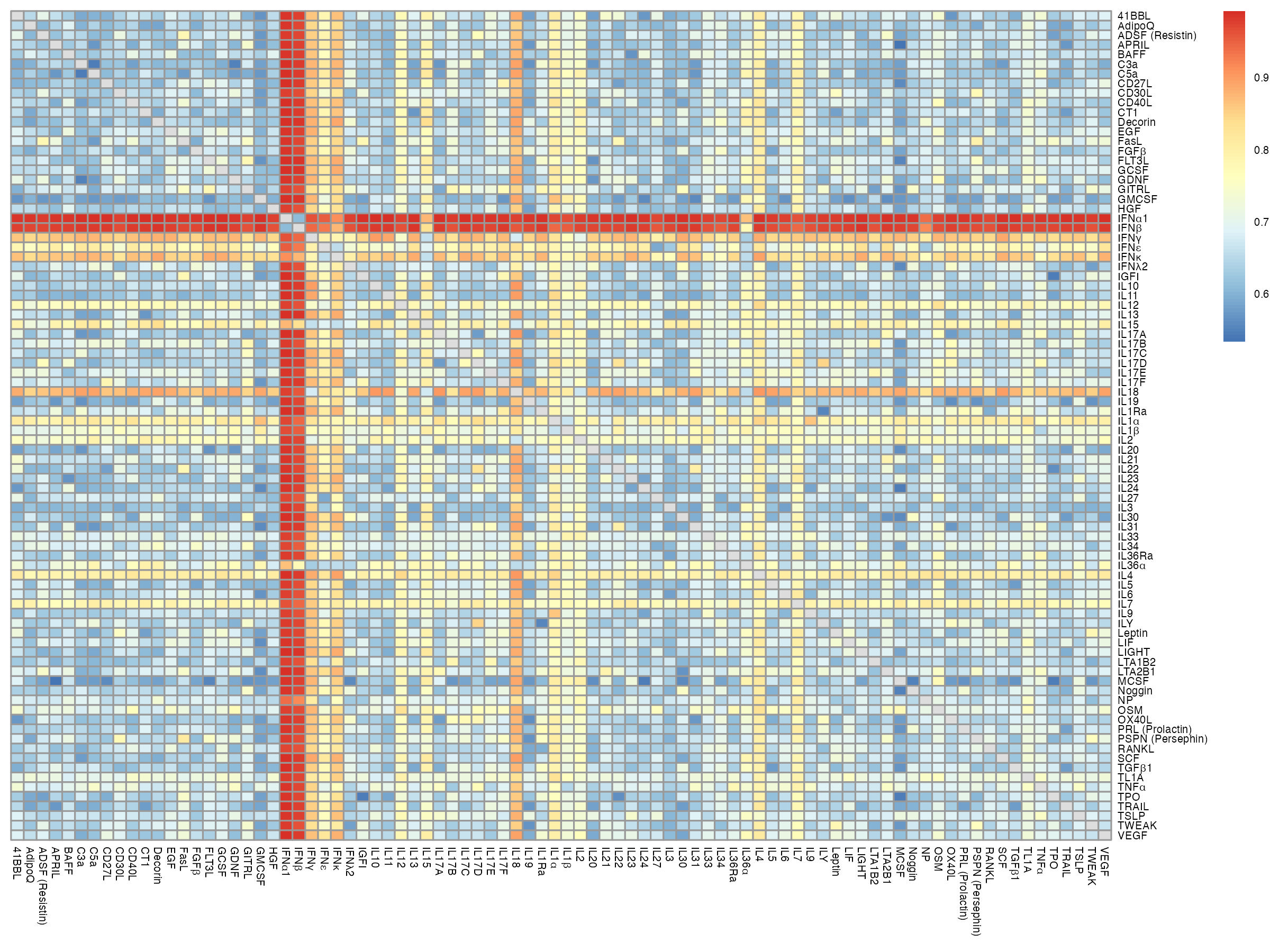

Supplement: S12 Fig — Rows represent cytokines to defined markers for (i.e., cytokines that are differentiated for). (TIFF) [file pcbi.1013475.s012.tiff]

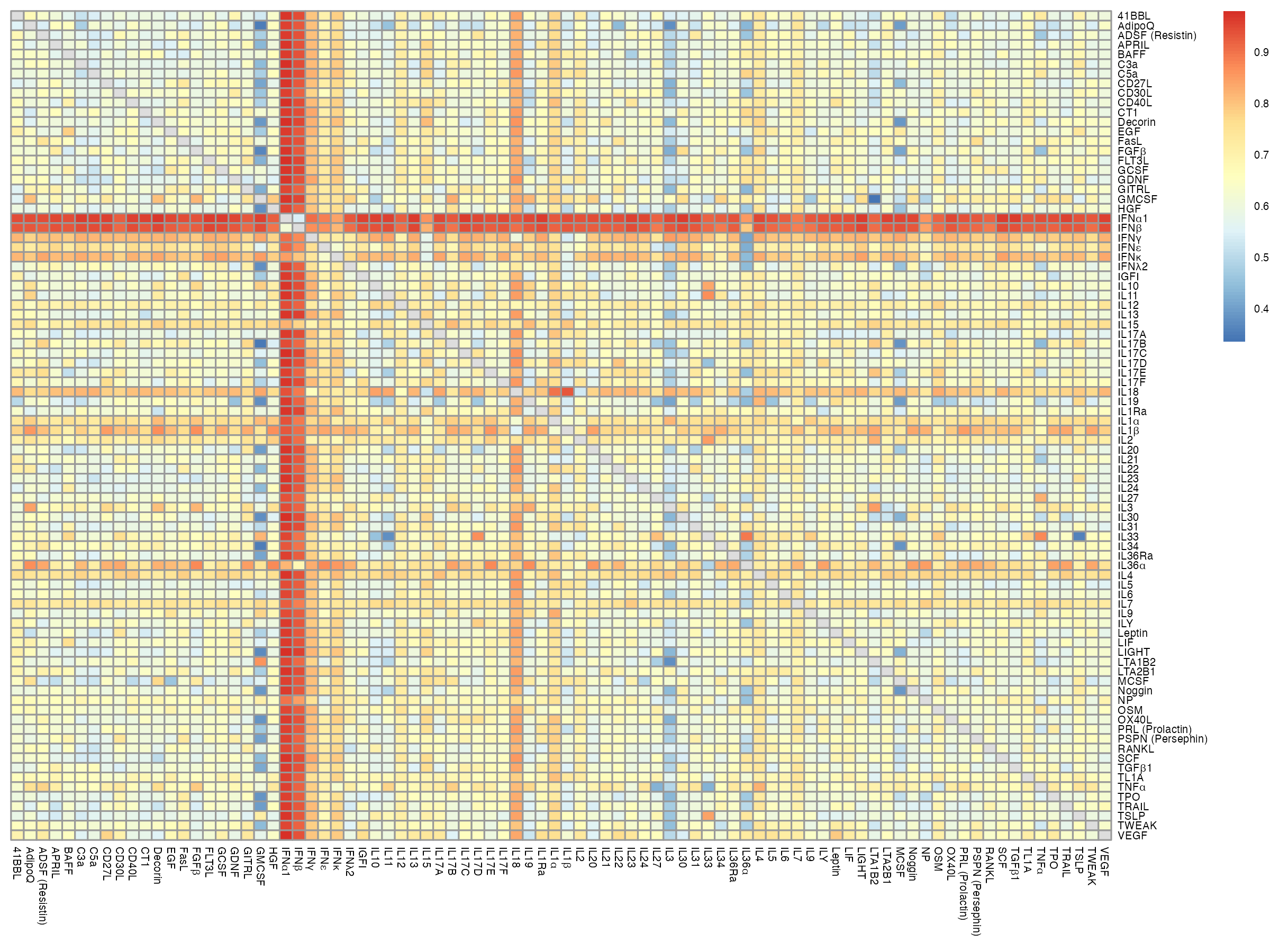

Supplement: S13 Fig — Rows represent cytokines to defined markers for (i.e., cytokines that are differentiated for). (TIFF) [file pcbi.1013475.s013.tiff]

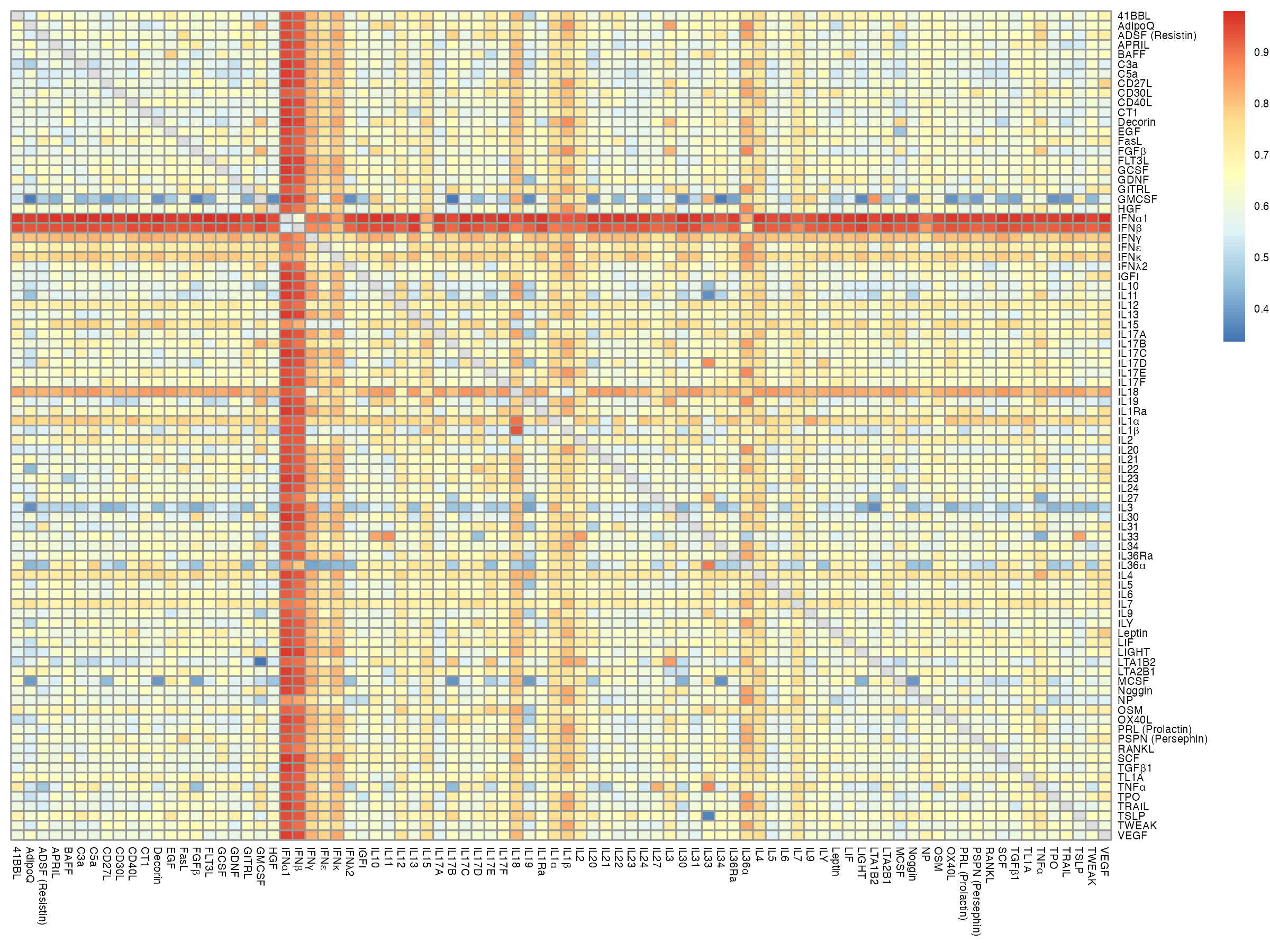

Supplement: S14 Fig — Rows represent cytokines to defined markers for (i.e., cytokines that are differentiated for). (TIFF) [file pcbi.1013475.s014.tiff]

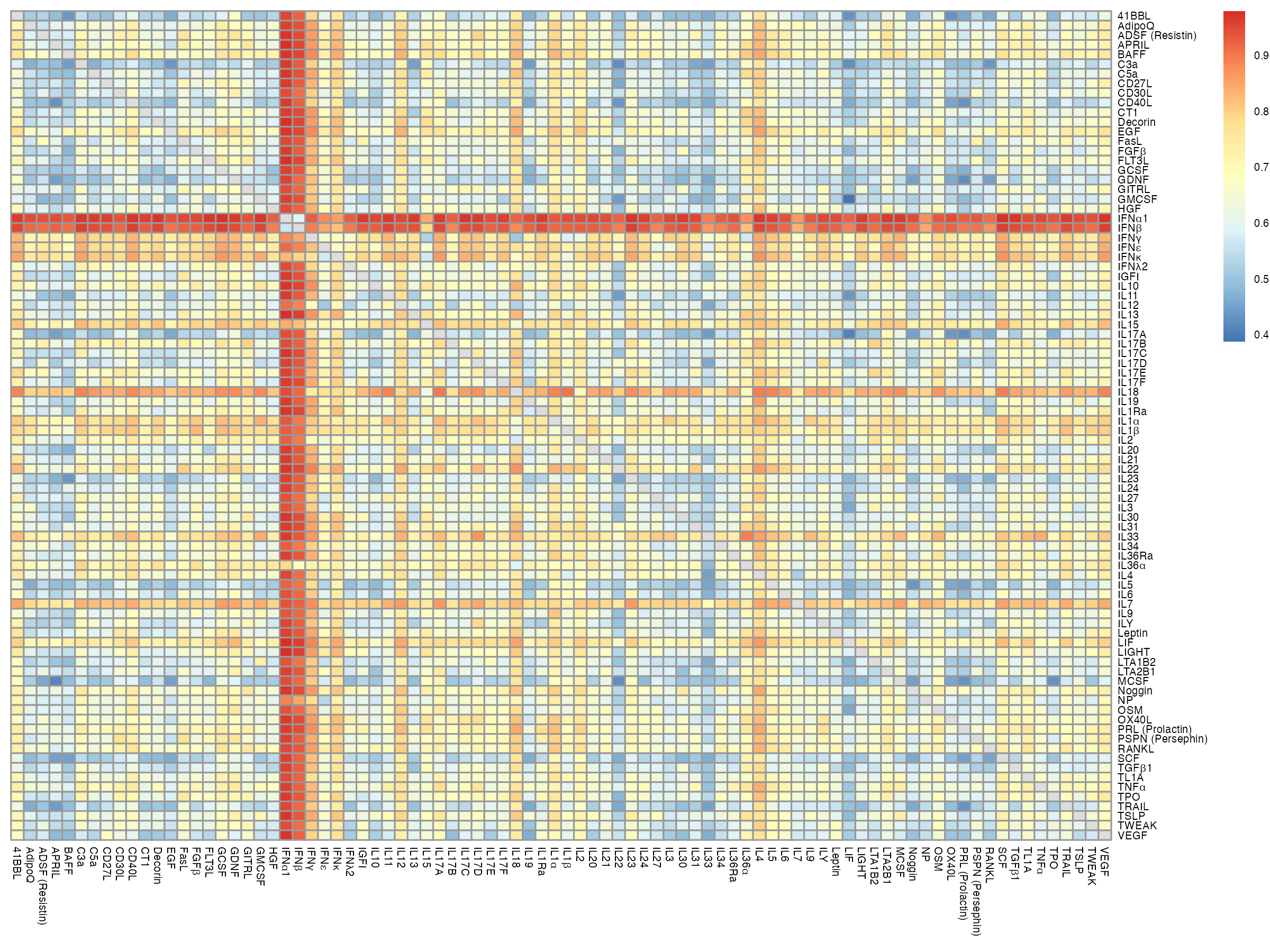

Supplement: S15 Fig — Rows represent cytokines to defined markers for (i.e., cytokines that are differentiated for). (TIFF) [file pcbi.1013475.s015.tiff]

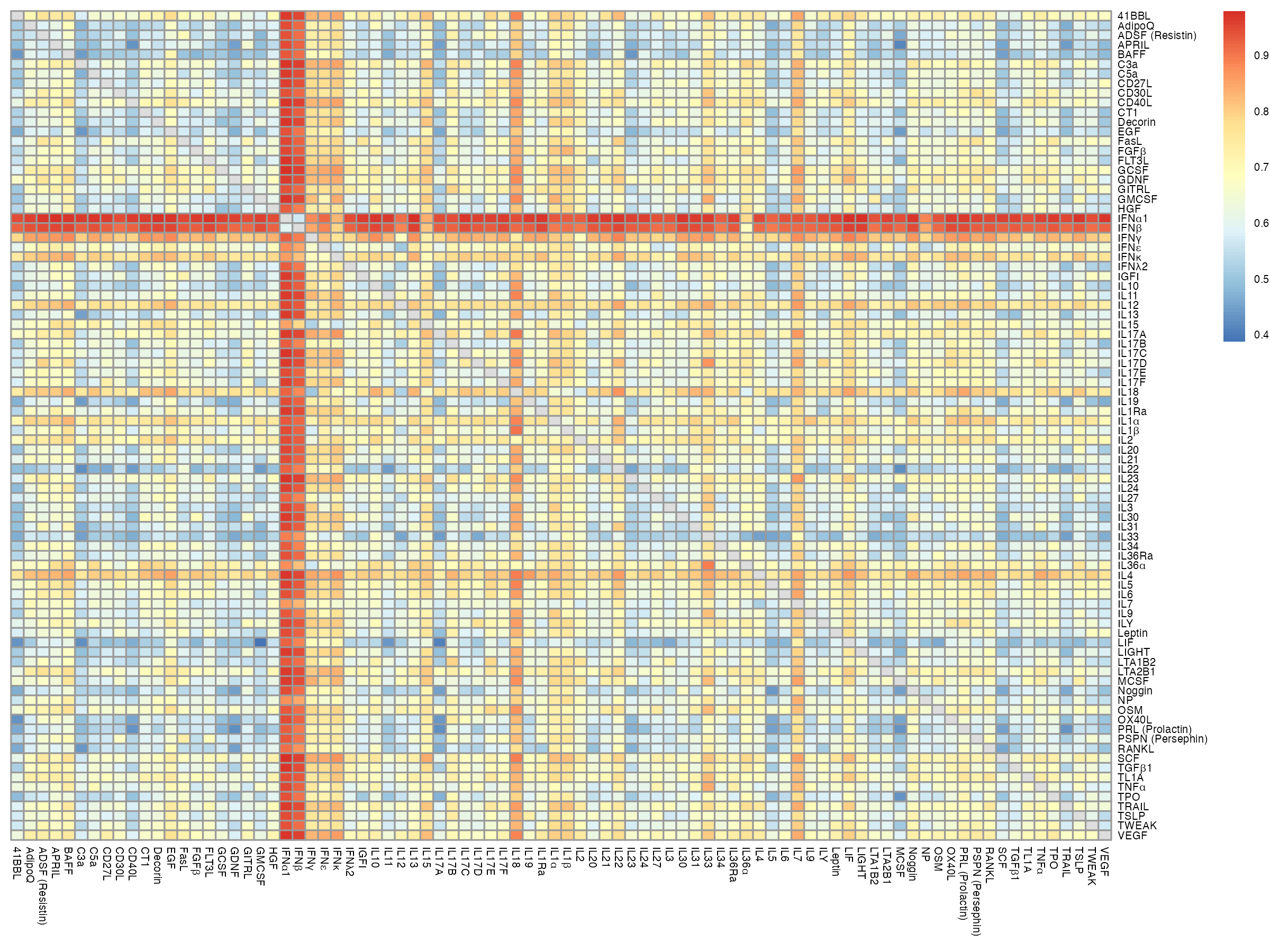

Supplement: S16 Fig — Rows represent cytokines to defined markers for (i.e., cytokines that are differentiated for). (TIFF) [file pcbi.1013475.s016.tiff]

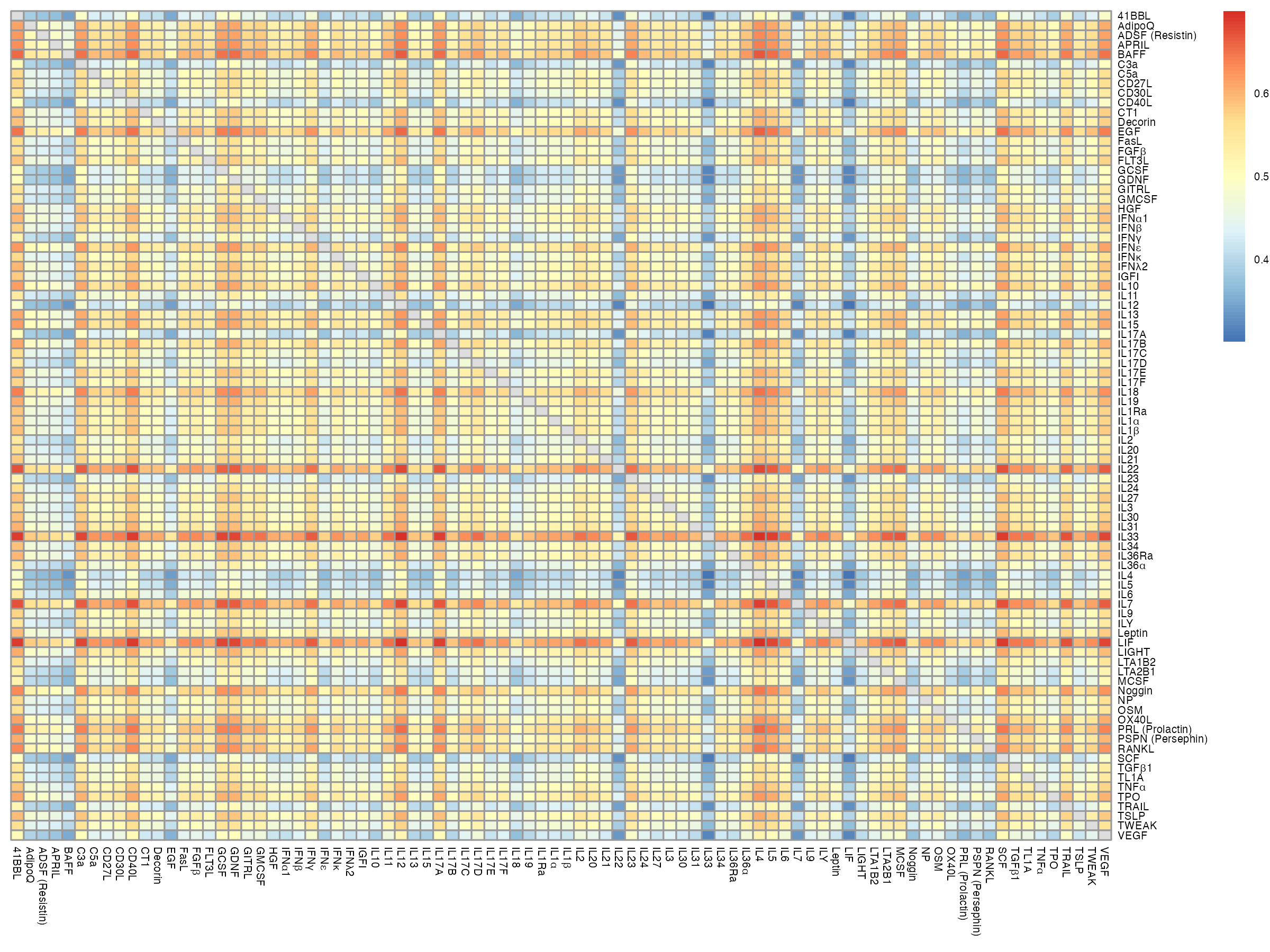

Supplement: S17 Fig — Rows represent cytokines to defined markers for (i.e., cytokines that are differentiated for). (TIFF) [file pcbi.1013475.s017.tiff]

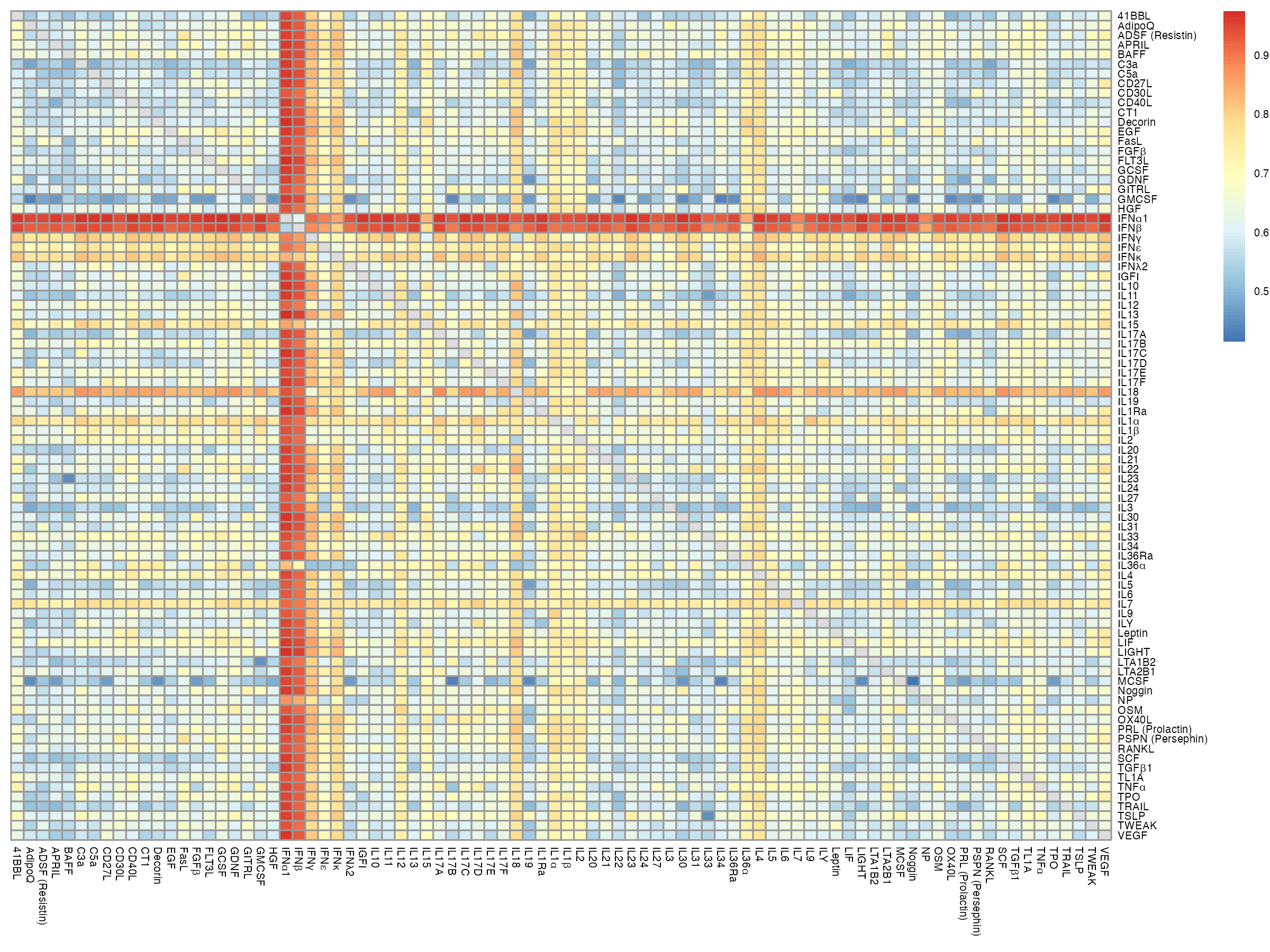

Supplement: S18 Fig — Rows represent cytokines to defined markers for (i.e., cytokines that are differentiated for). (TIFF) [file pcbi.1013475.s018.tiff]

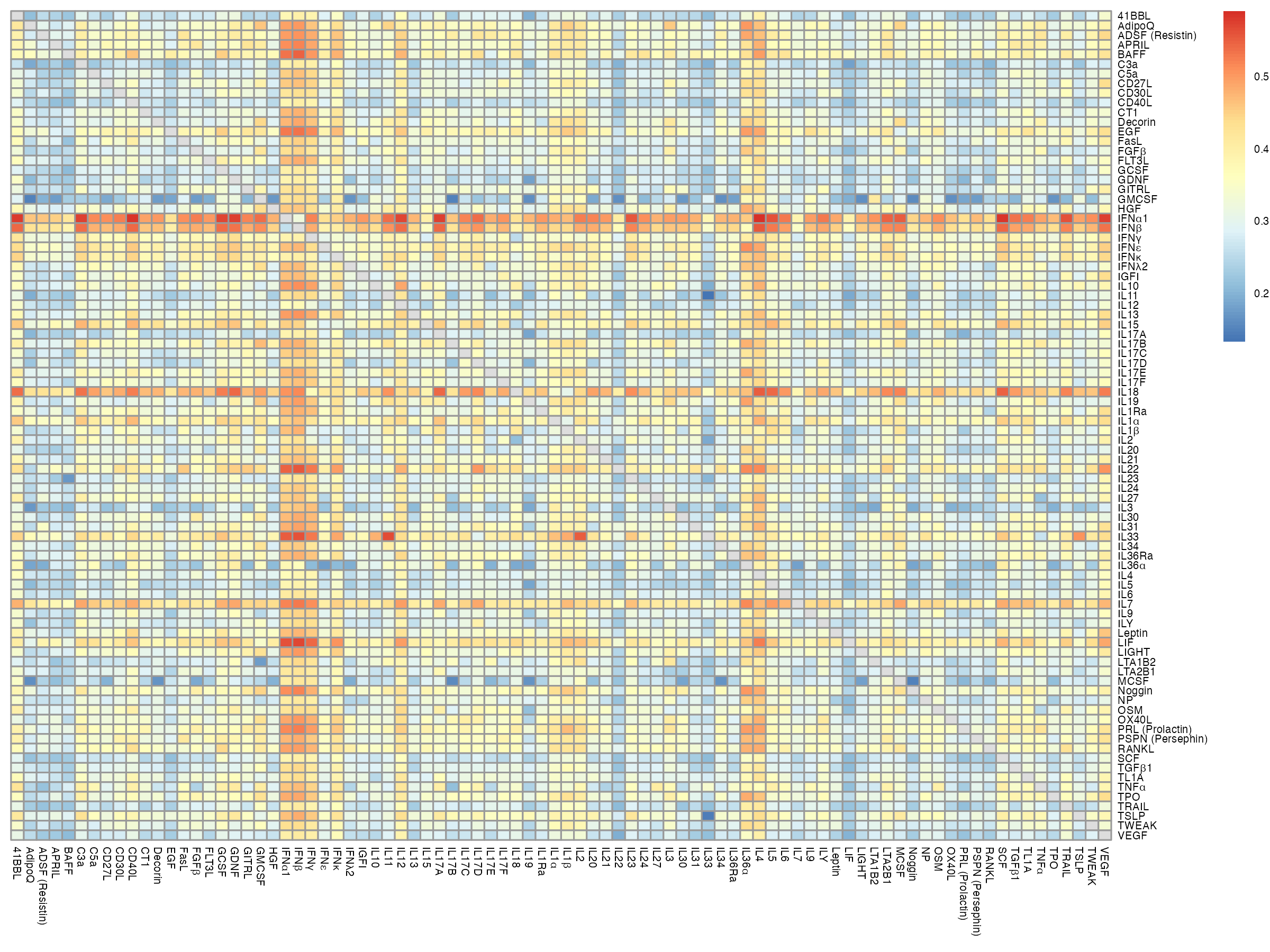

Supplement: S19 Fig — Rows represent cytokines to defined markers for (i.e., cytokines that are differentiated for). (TIFF) [file pcbi.1013475.s019.tiff]

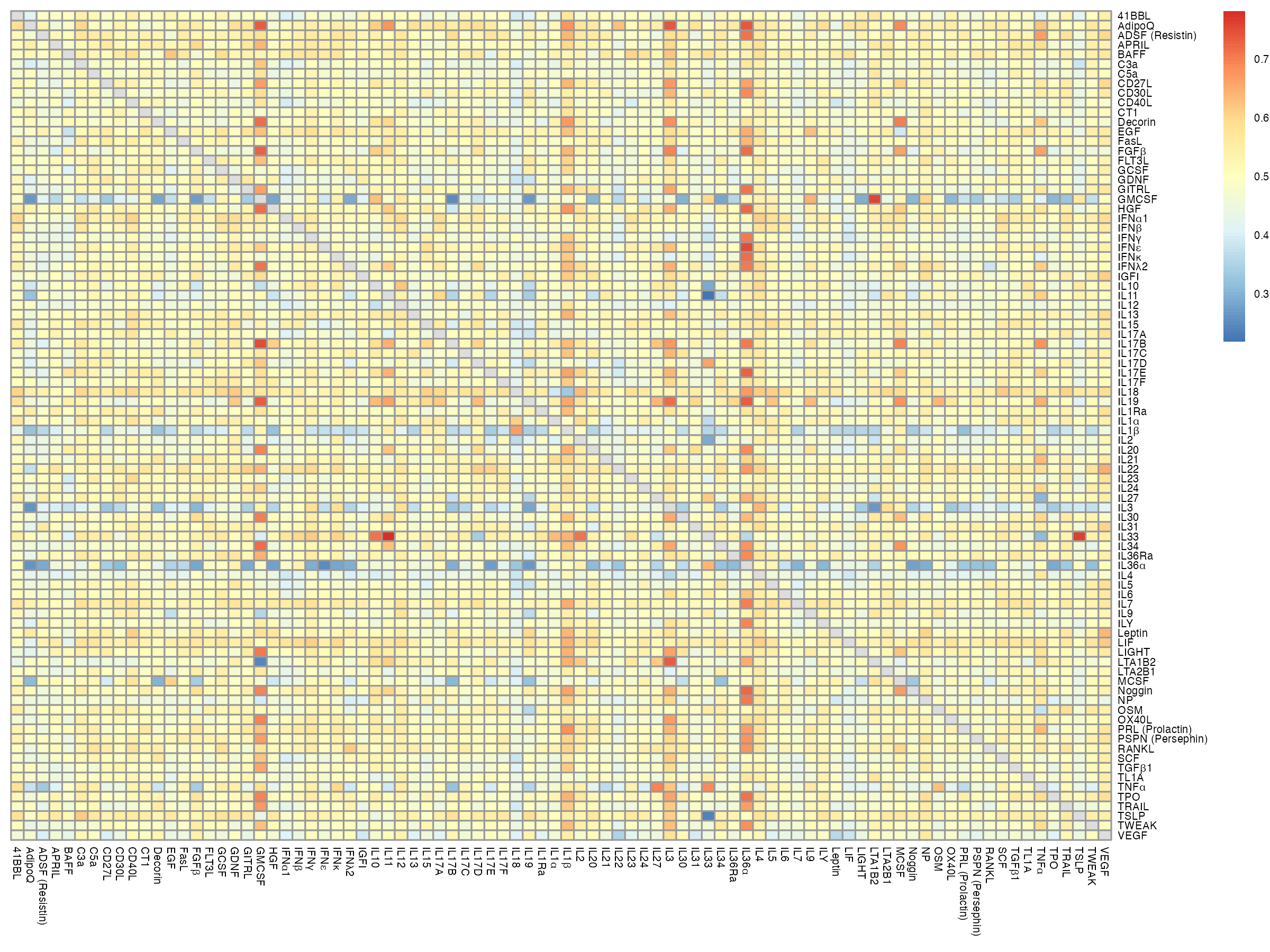

Supplement: S20 Fig — Rows represent cytokines to defined markers for (i.e., cytokines that are differentiated for). (TIFF) [file pcbi.1013475.s020.tiff]

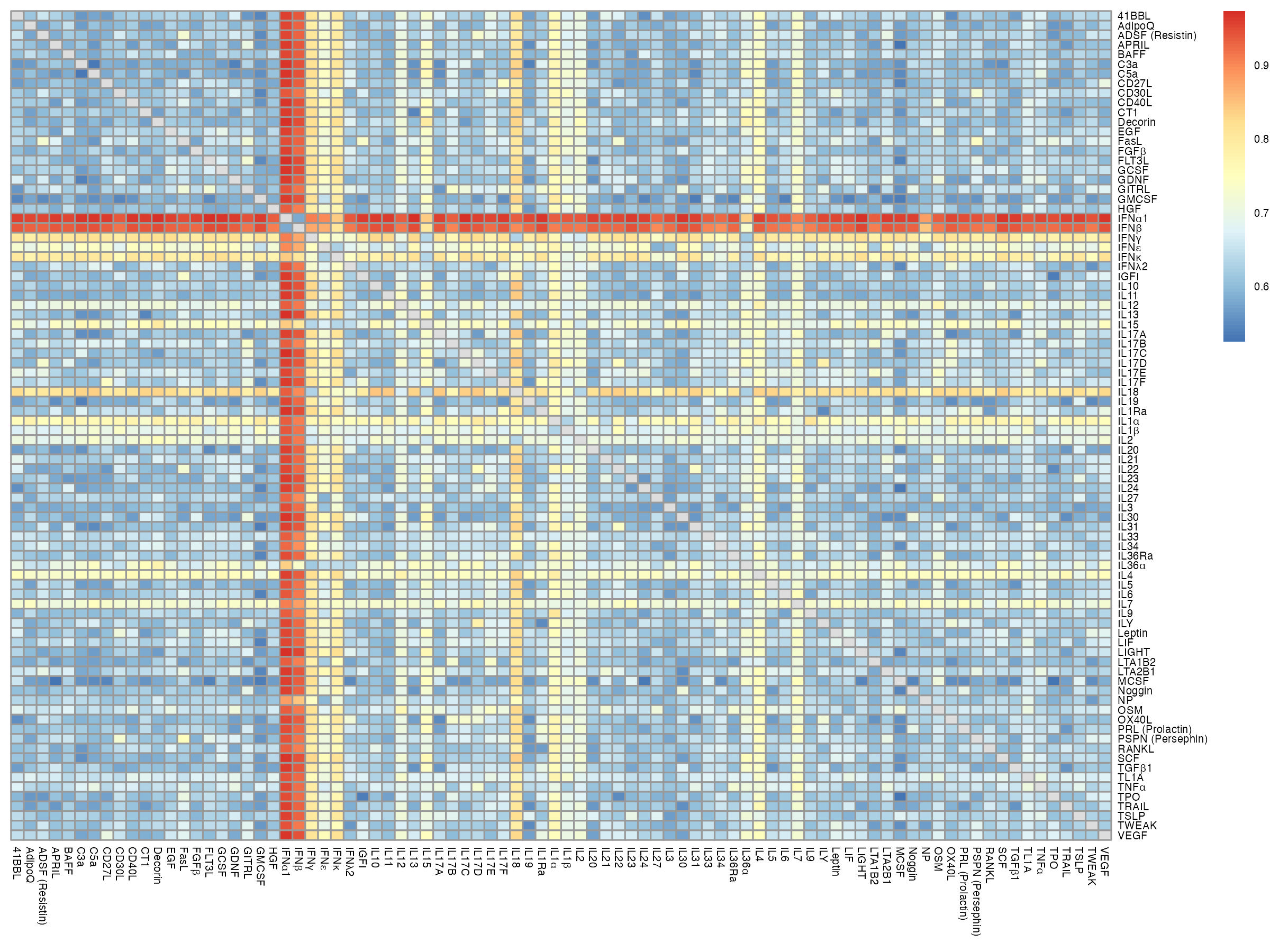

Supplement: S21 Fig — Rows represent cytokines to defined markers for (i.e., cytokines that are differentiated for). (TIFF) [file pcbi.1013475.s021.tiff]

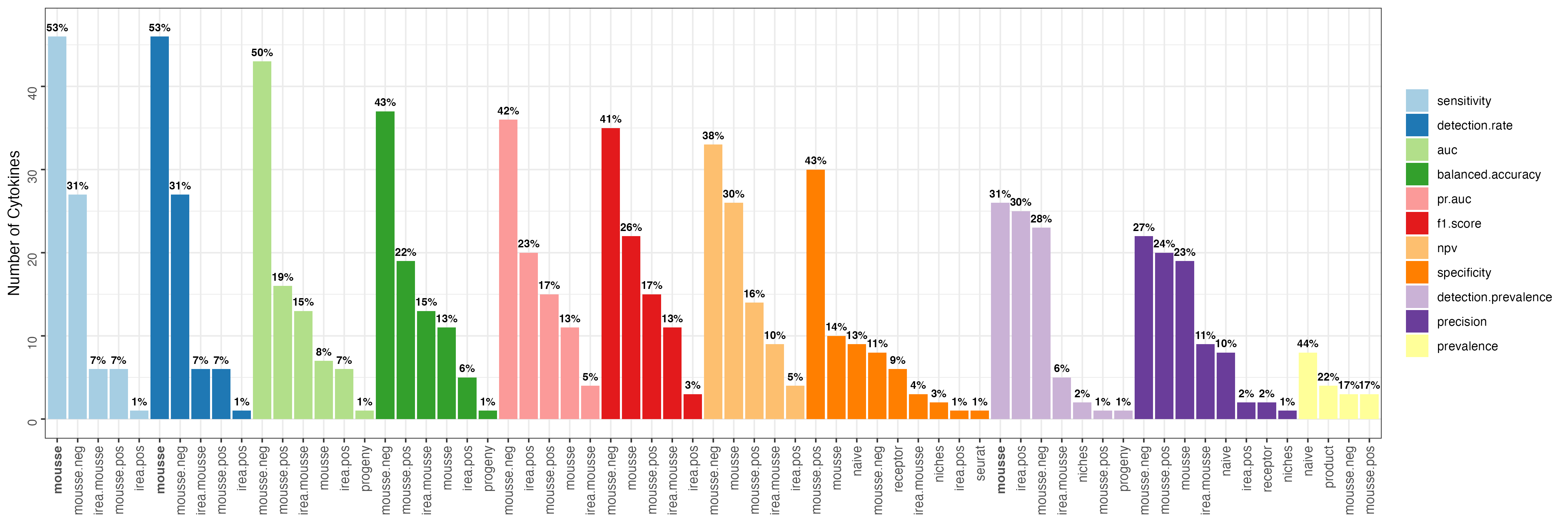

Supplement: S22 Fig — (TIFF) [file pcbi.1013475.s022.tiff]

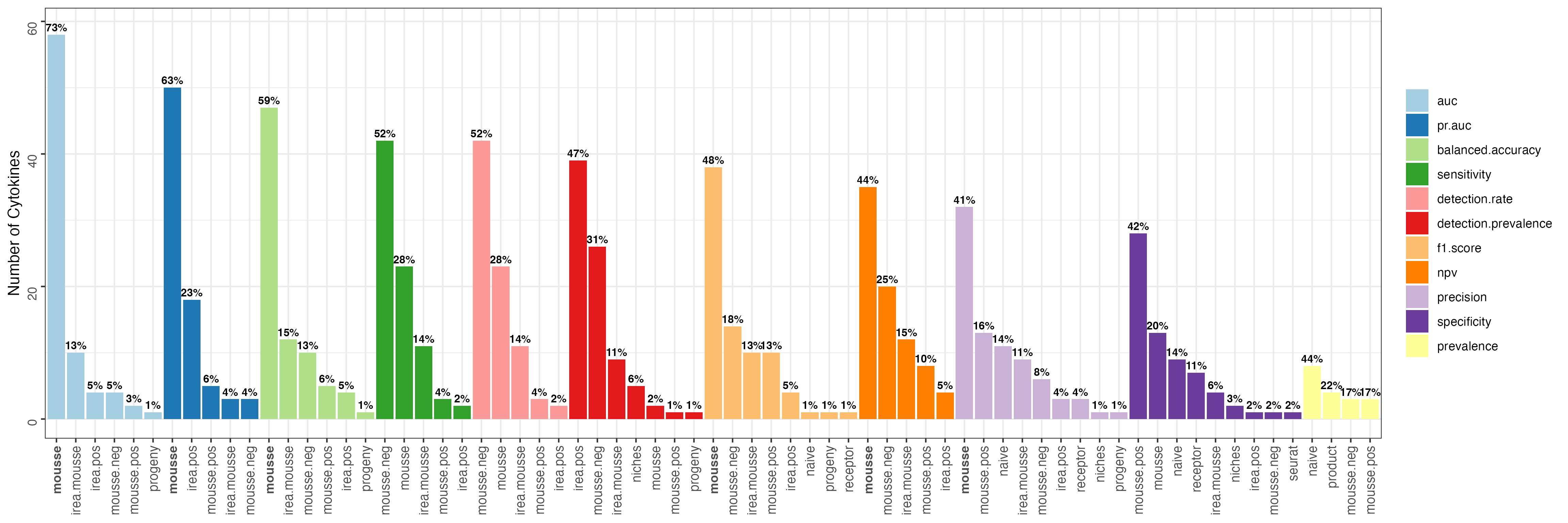

Supplement: S23 Fig — (TIFF) [file pcbi.1013475.s023.tiff]

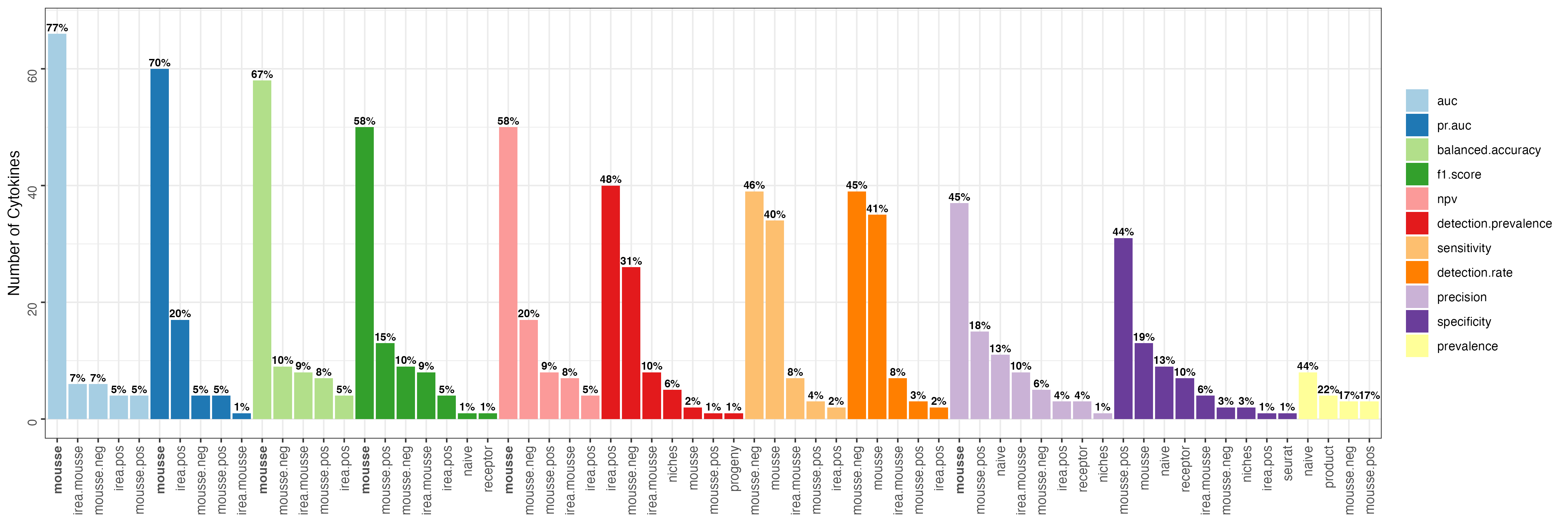

Supplement: S24 Fig — (TIFF) [file pcbi.1013475.s024.tiff]

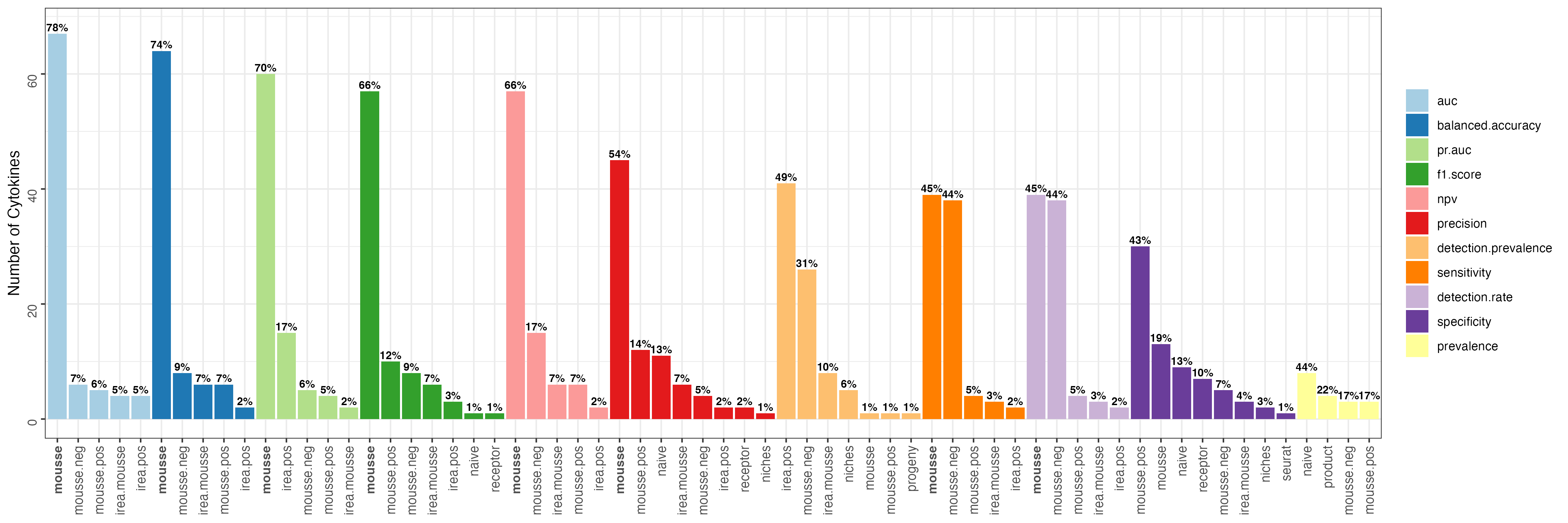

Supplement: S25 Fig — (TIFF) [file pcbi.1013475.s025.tiff]

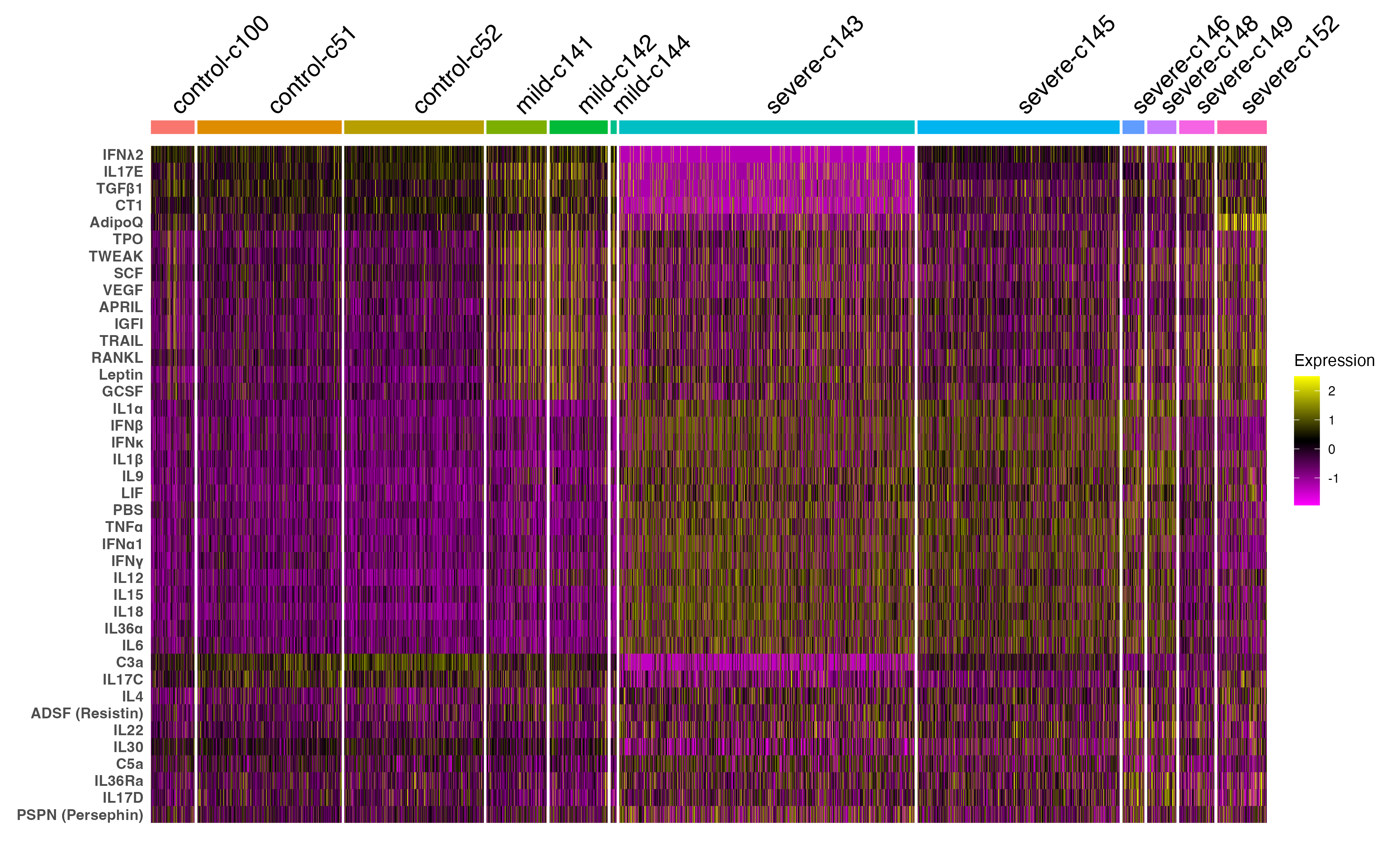

Supplement: S26 Fig — (TIFF) [file pcbi.1013475.s026.tiff]

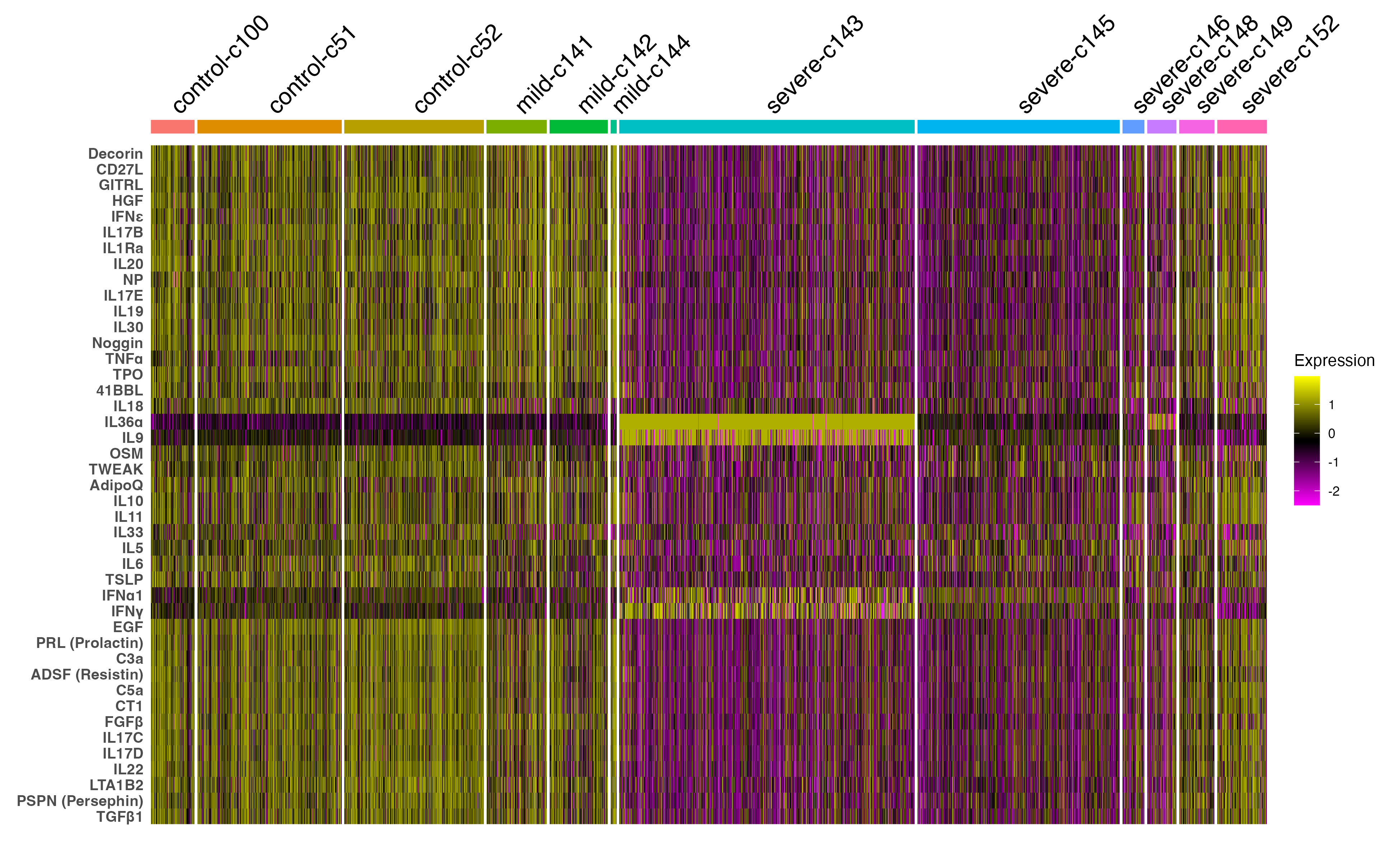

Supplement: S27 Fig — (TIFF) [file pcbi.1013475.s027.tiff]

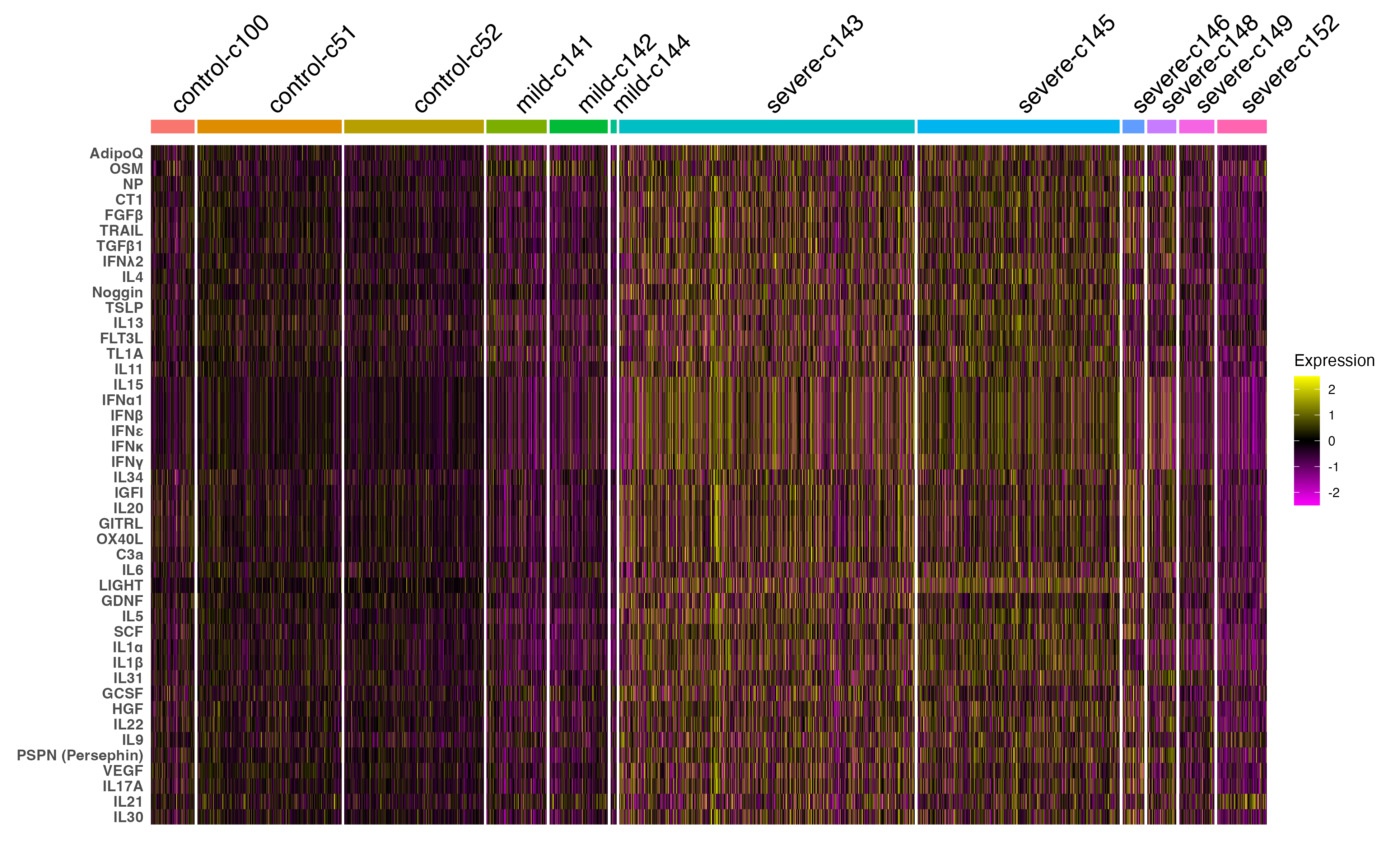

Supplement: S28 Fig — (TIFF) [file pcbi.1013475.s028.tiff]

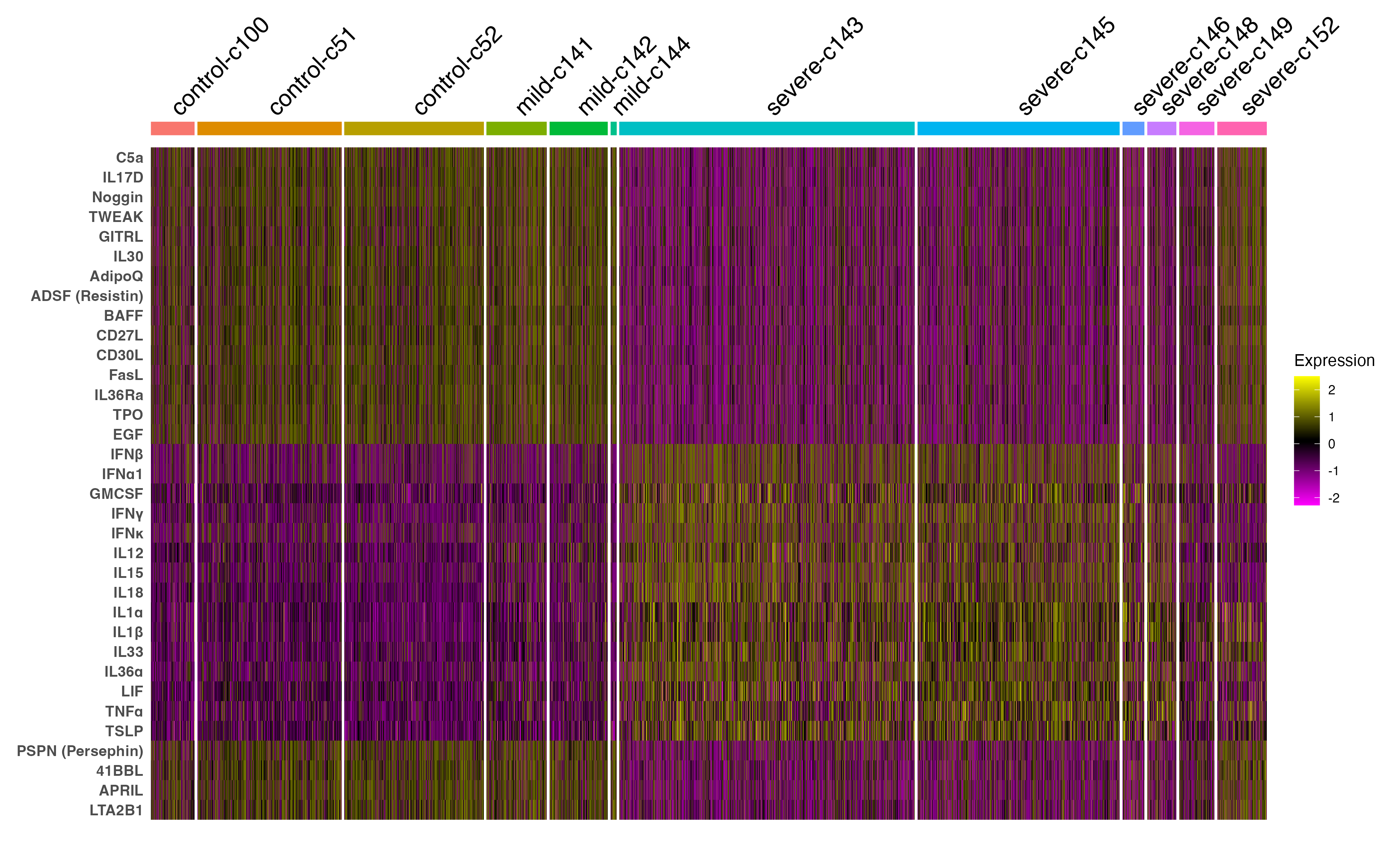

Supplement: S29 Fig — (TIFF) [file pcbi.1013475.s029.tiff]

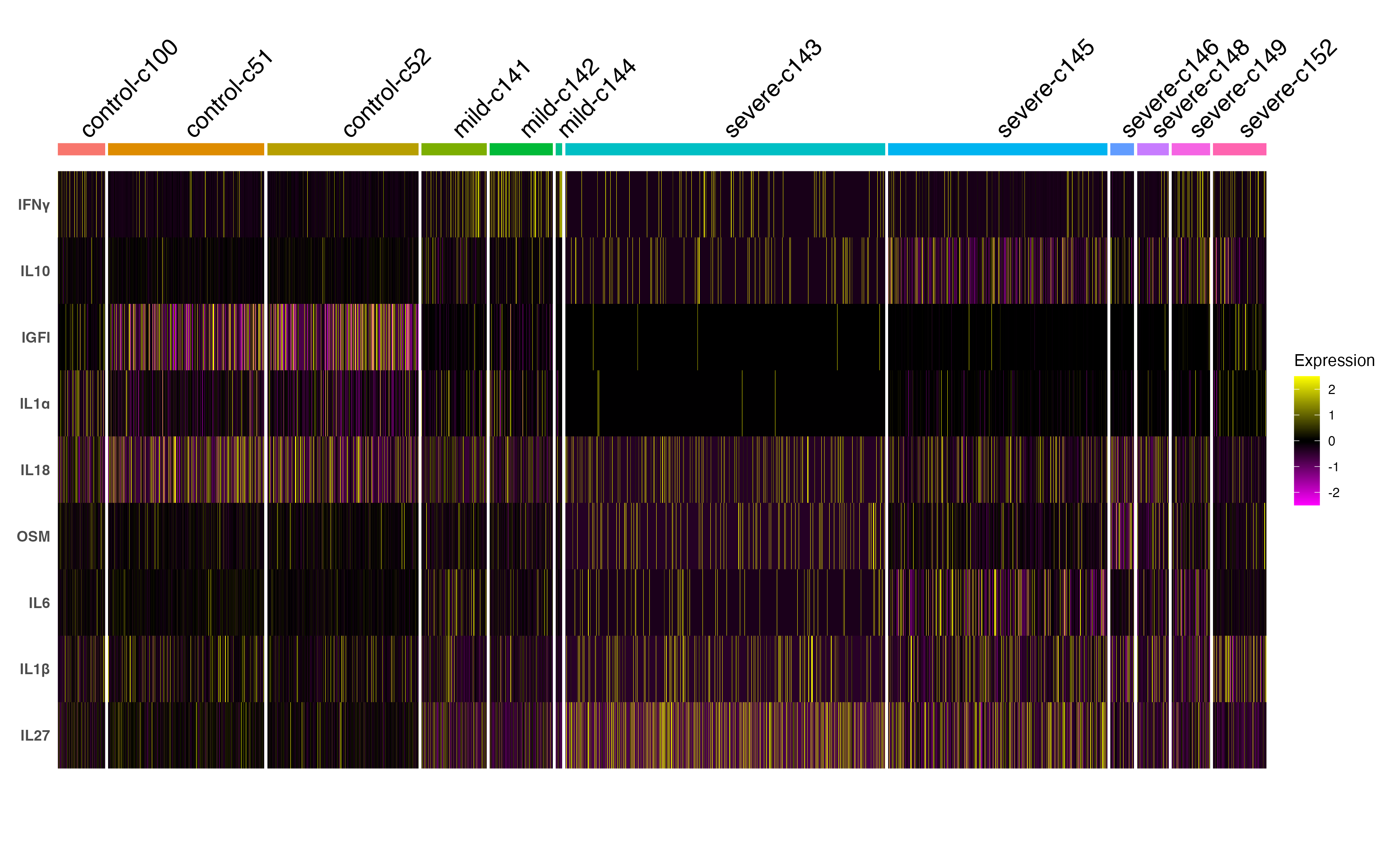

Supplement: S30 Fig — (TIFF) [file pcbi.1013475.s030.tiff]

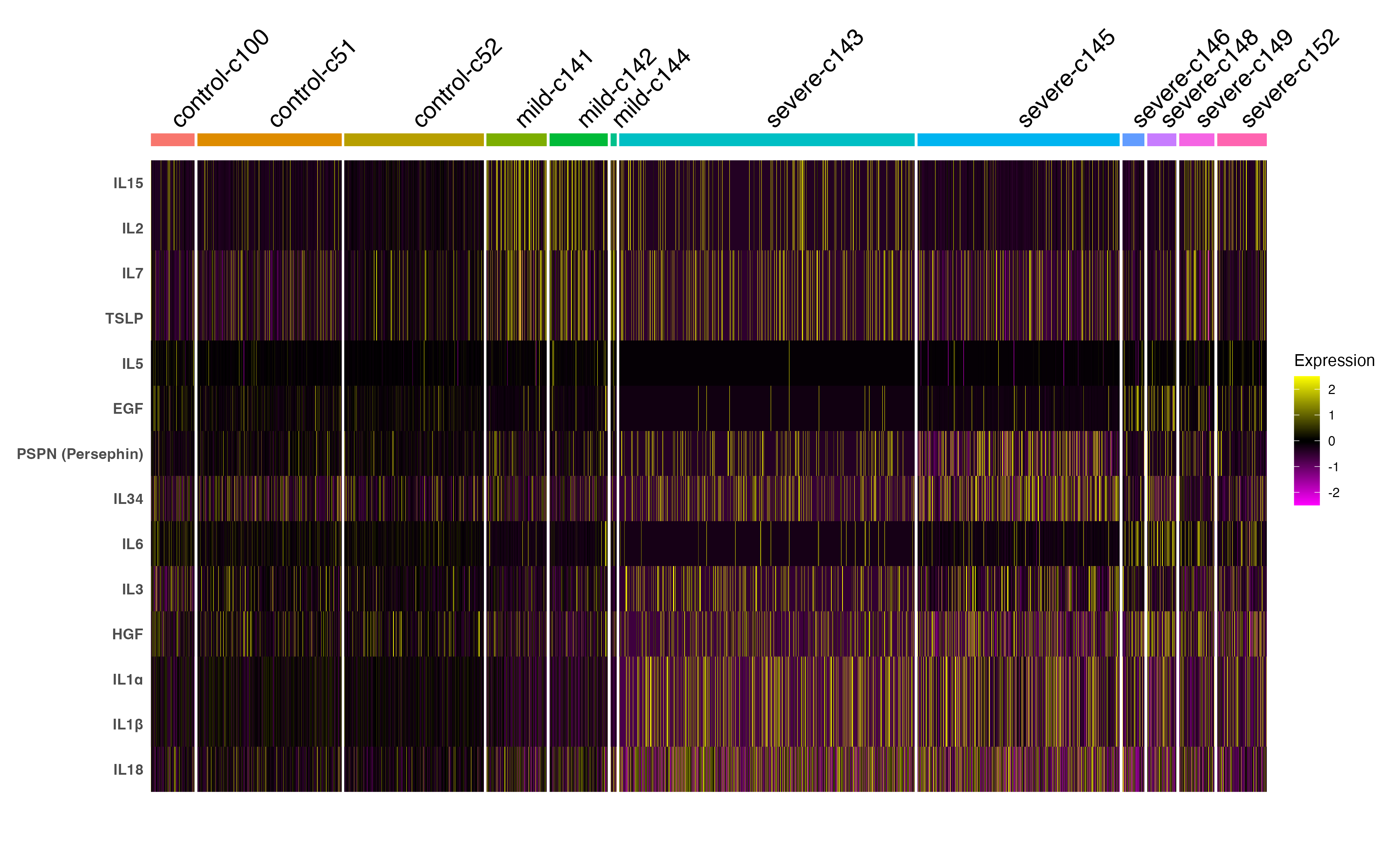

Supplement: S31 Fig — (TIFF) [file pcbi.1013475.s031.tiff]

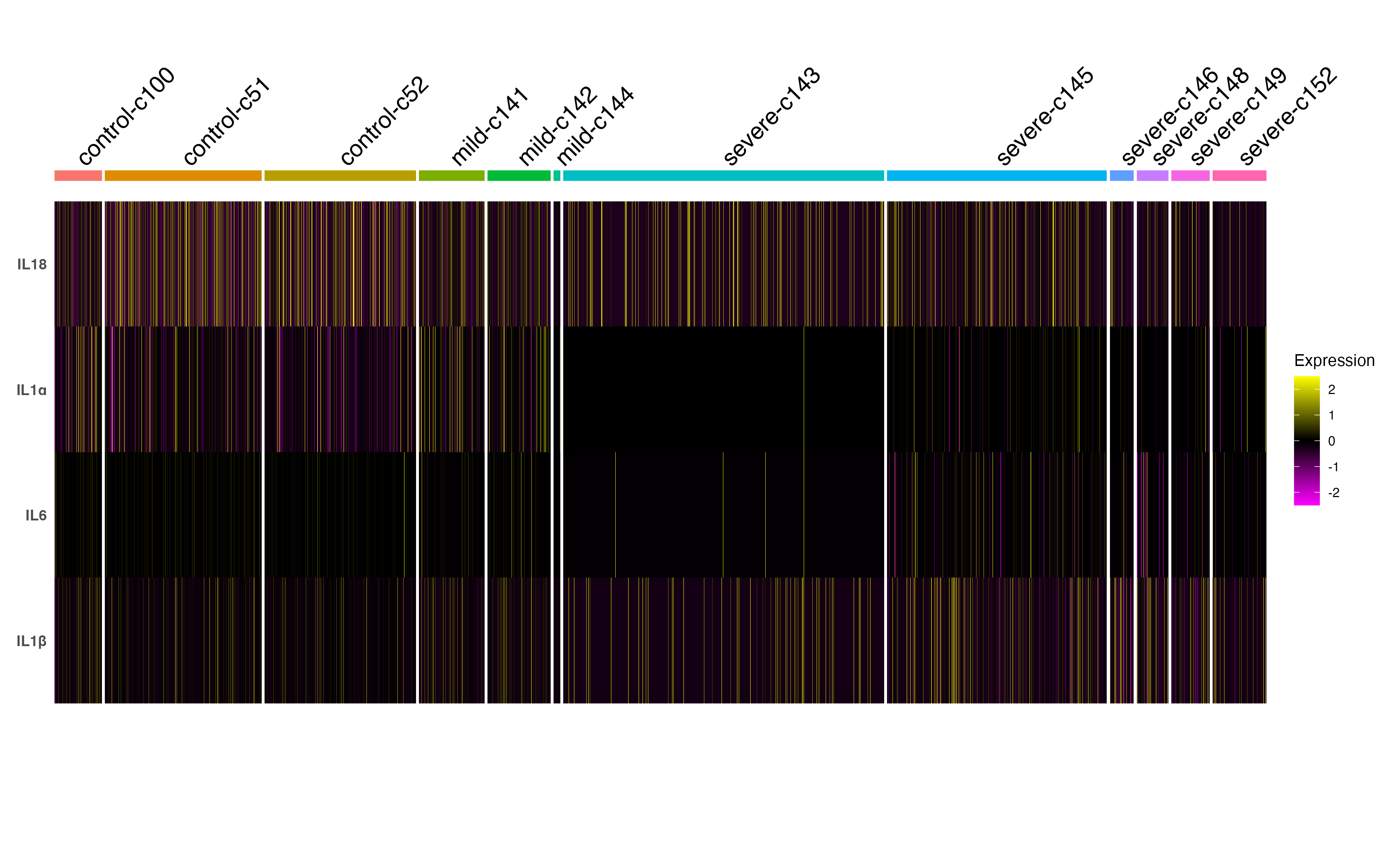

Supplement: S32 Fig — (TIFF) [file pcbi.1013475.s032.tiff]

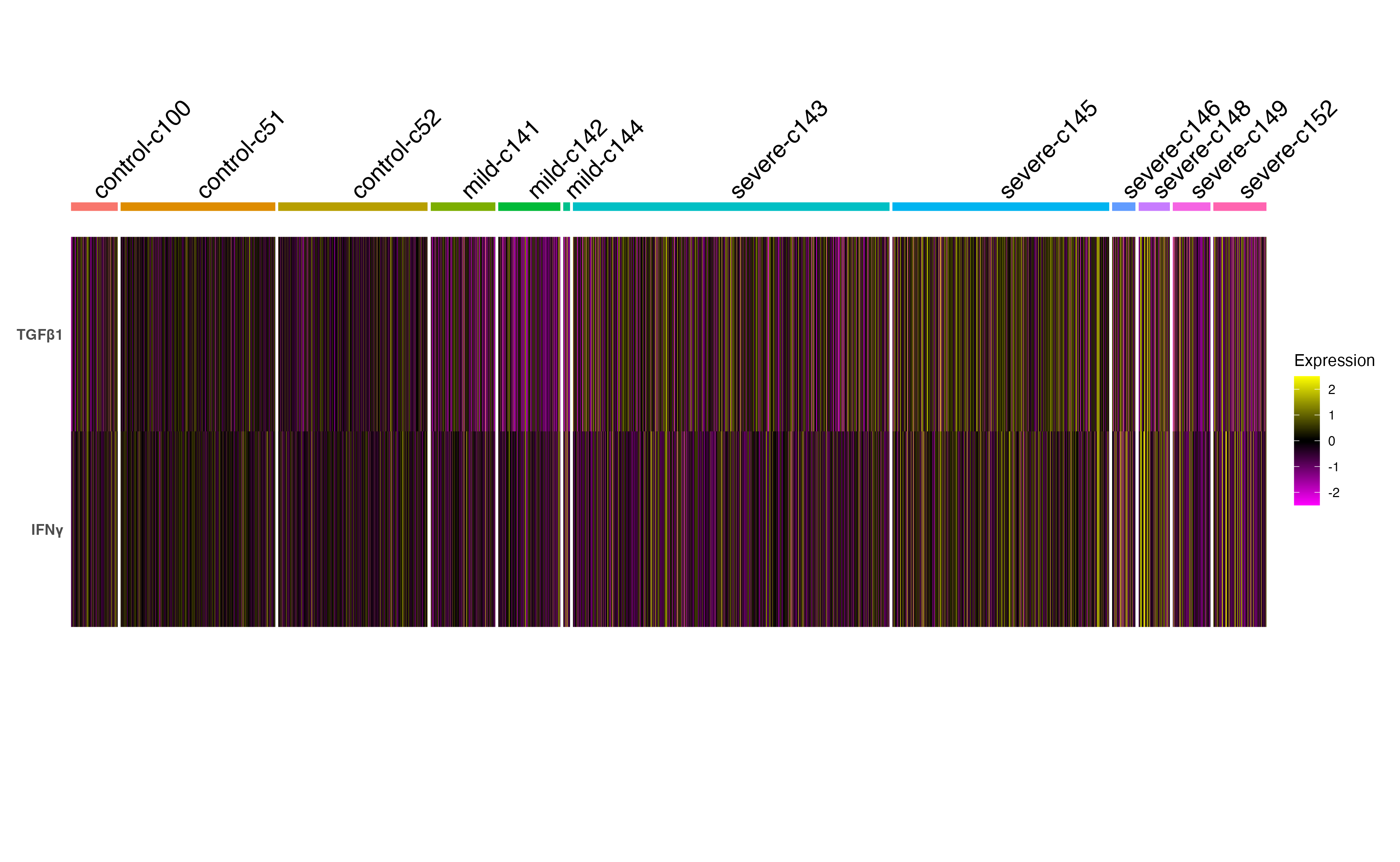

Supplement: S33 Fig — (TIFF) [file pcbi.1013475.s033.tiff]

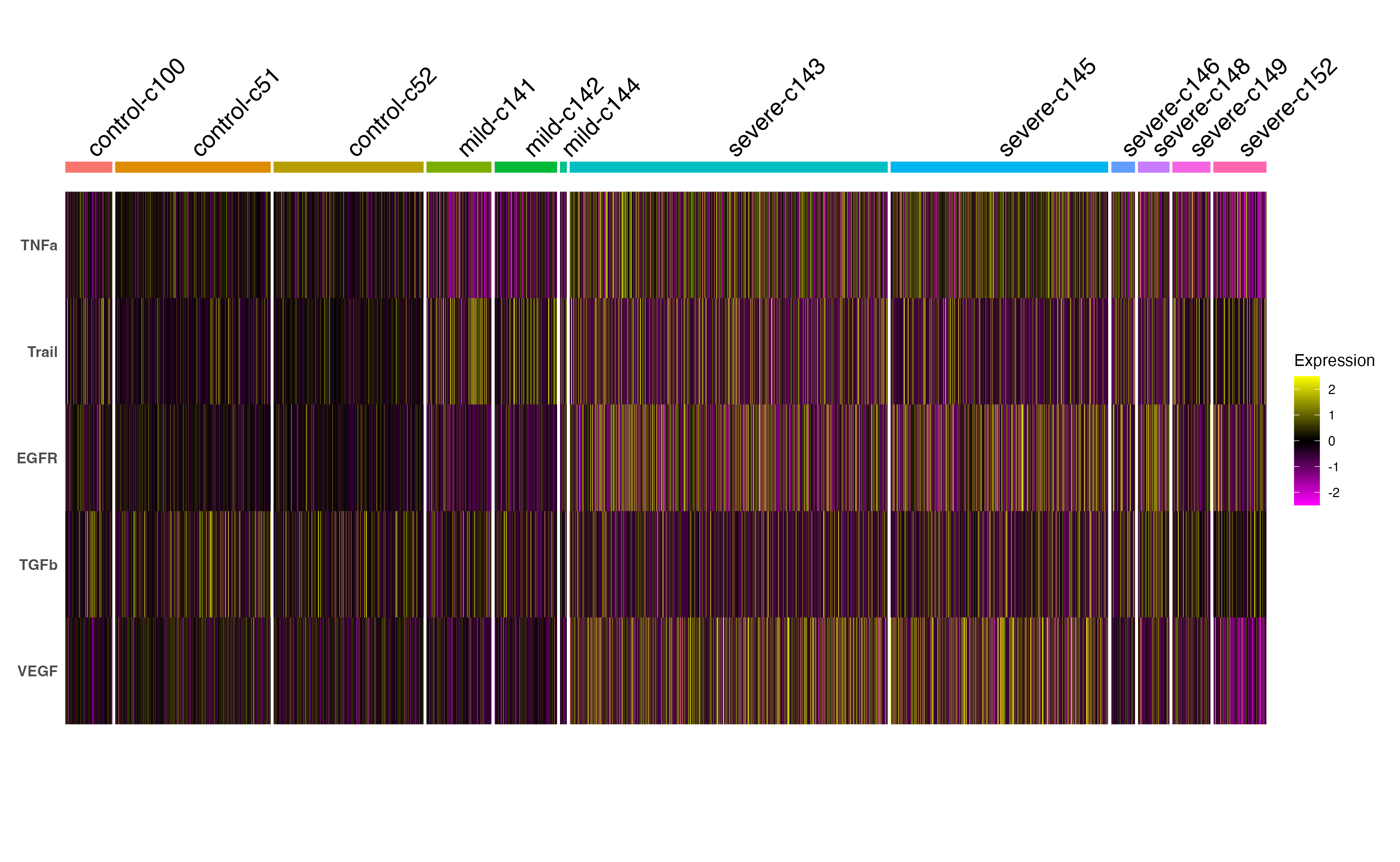

Supplement: S34 Fig — (TIFF) [file pcbi.1013475.s034.tiff]

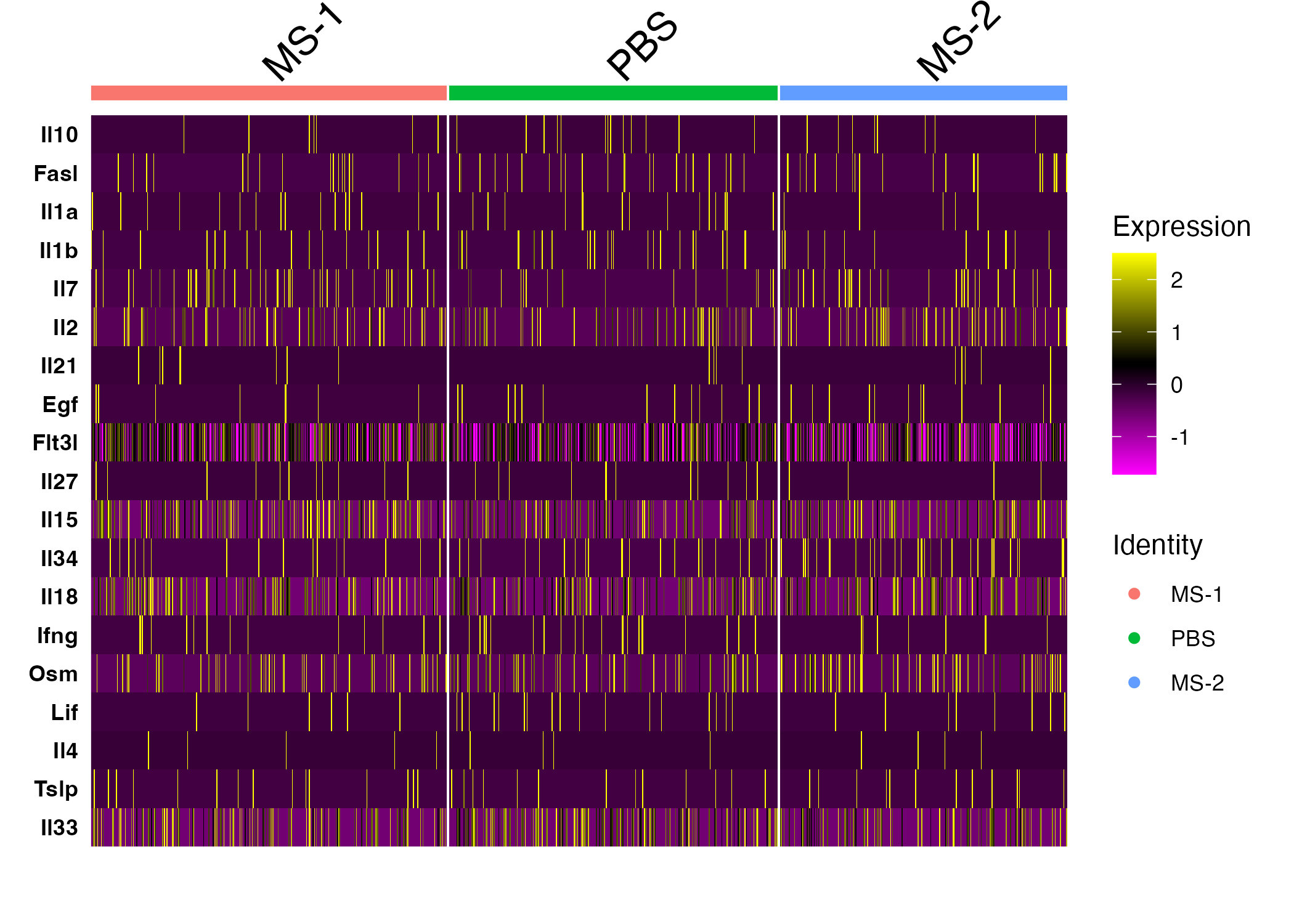

Supplement: S35 Fig — (TIFF) [file pcbi.1013475.s035.tiff]

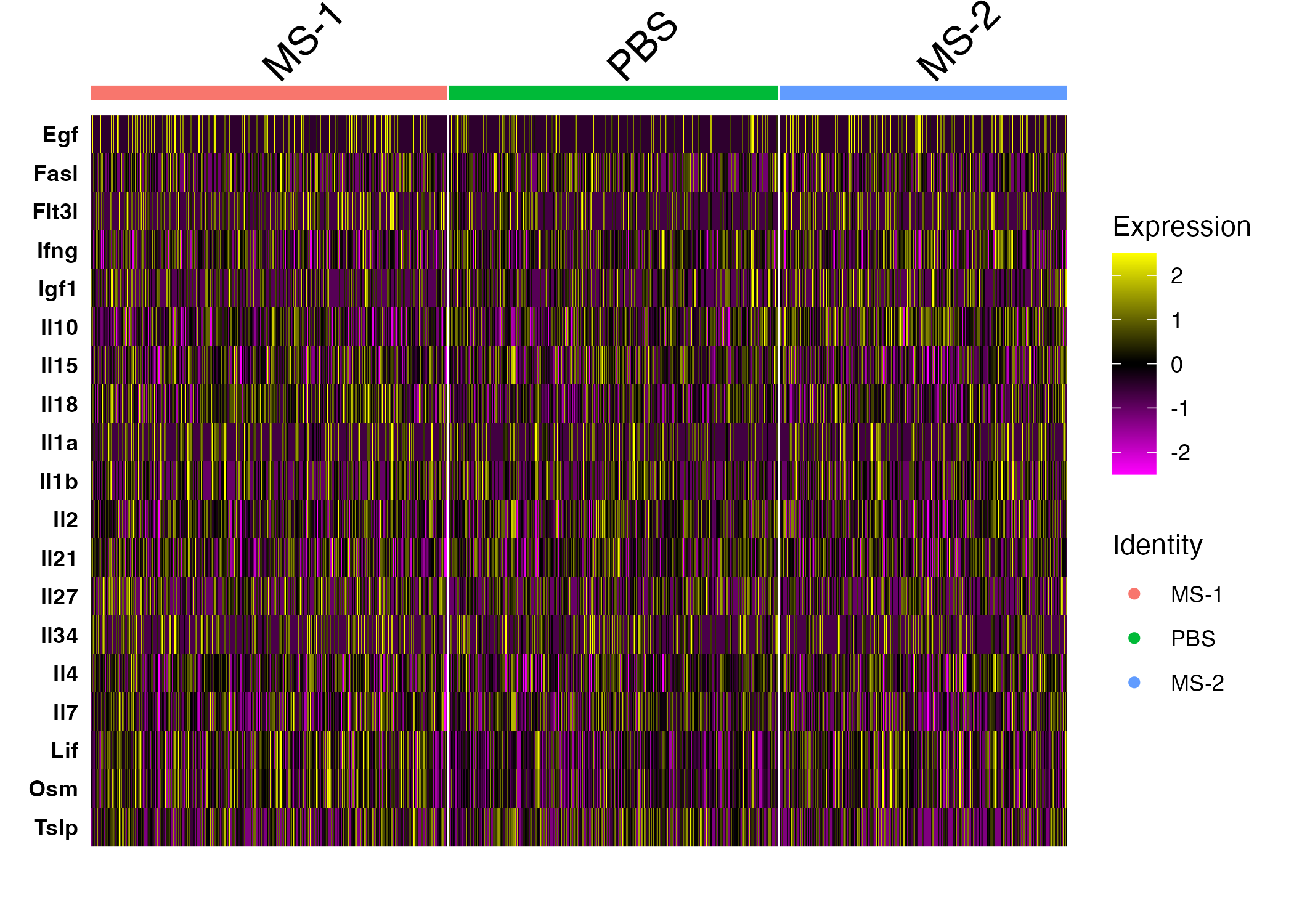

Supplement: S36 Fig — (TIFF) [file pcbi.1013475.s036.tiff]

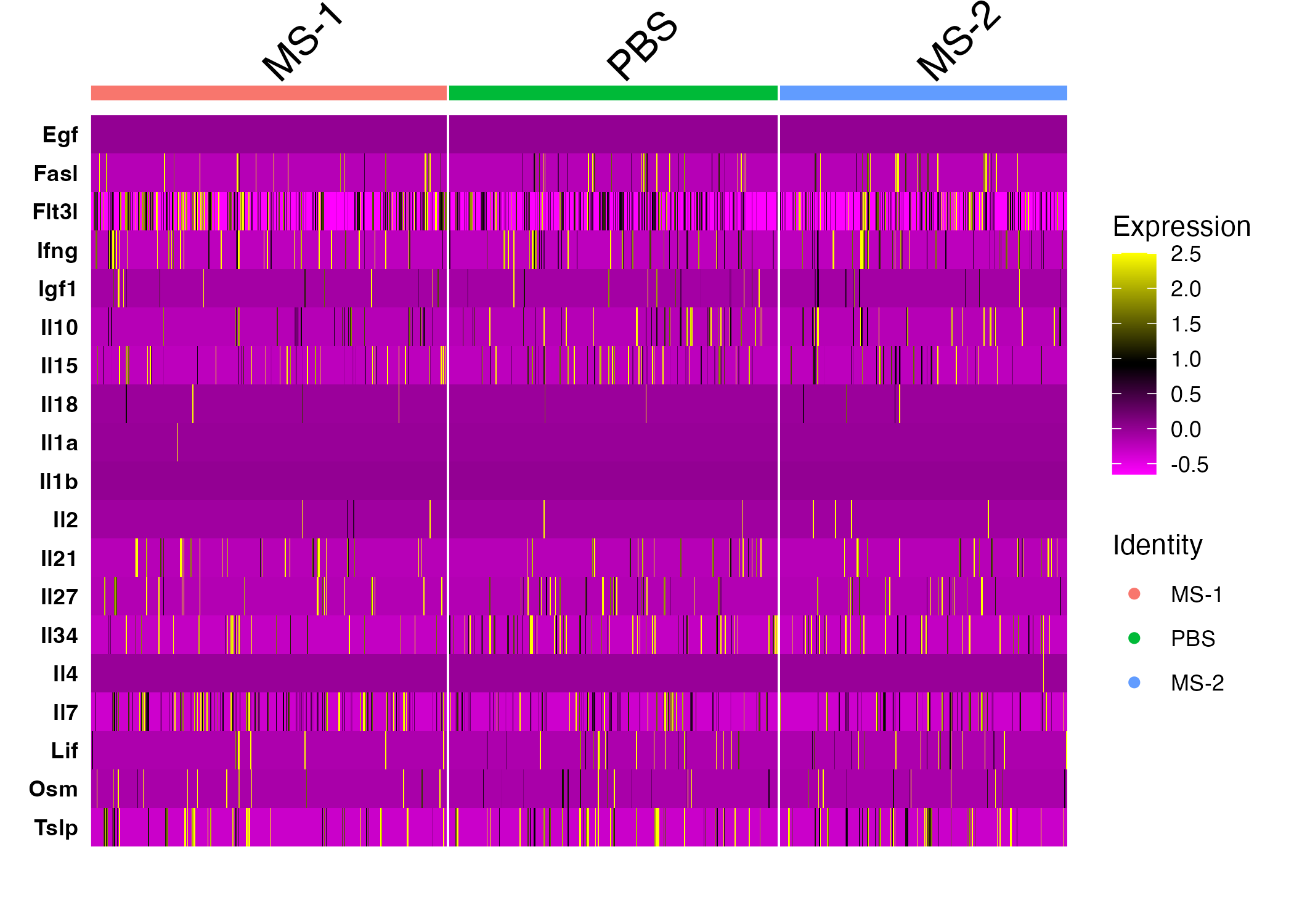

Supplement: S38 Fig — (TIFF) [file pcbi.1013475.s038.tiff]

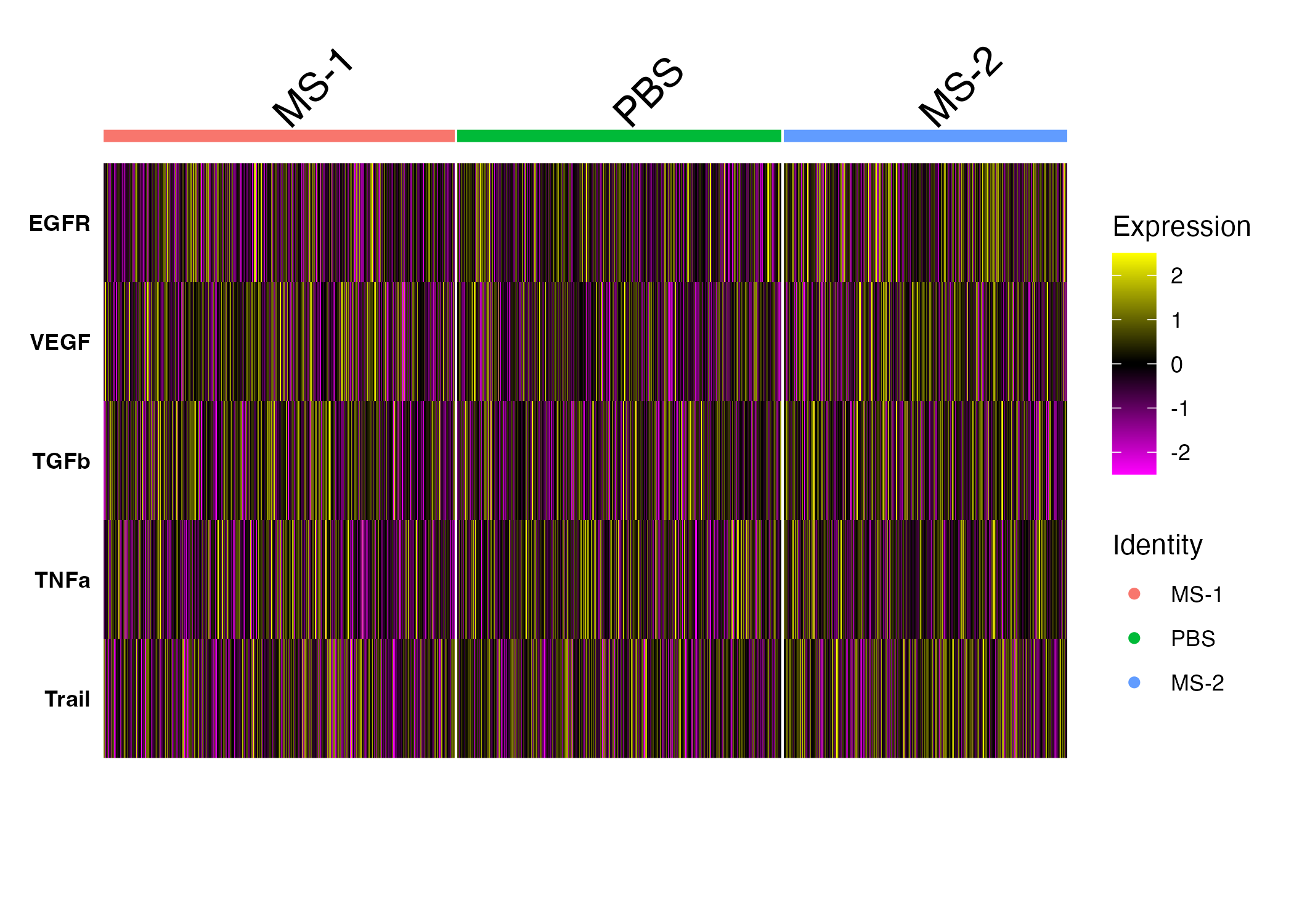

Supplement: S39 Fig — (TIFF) [file pcbi.1013475.s039.tiff]

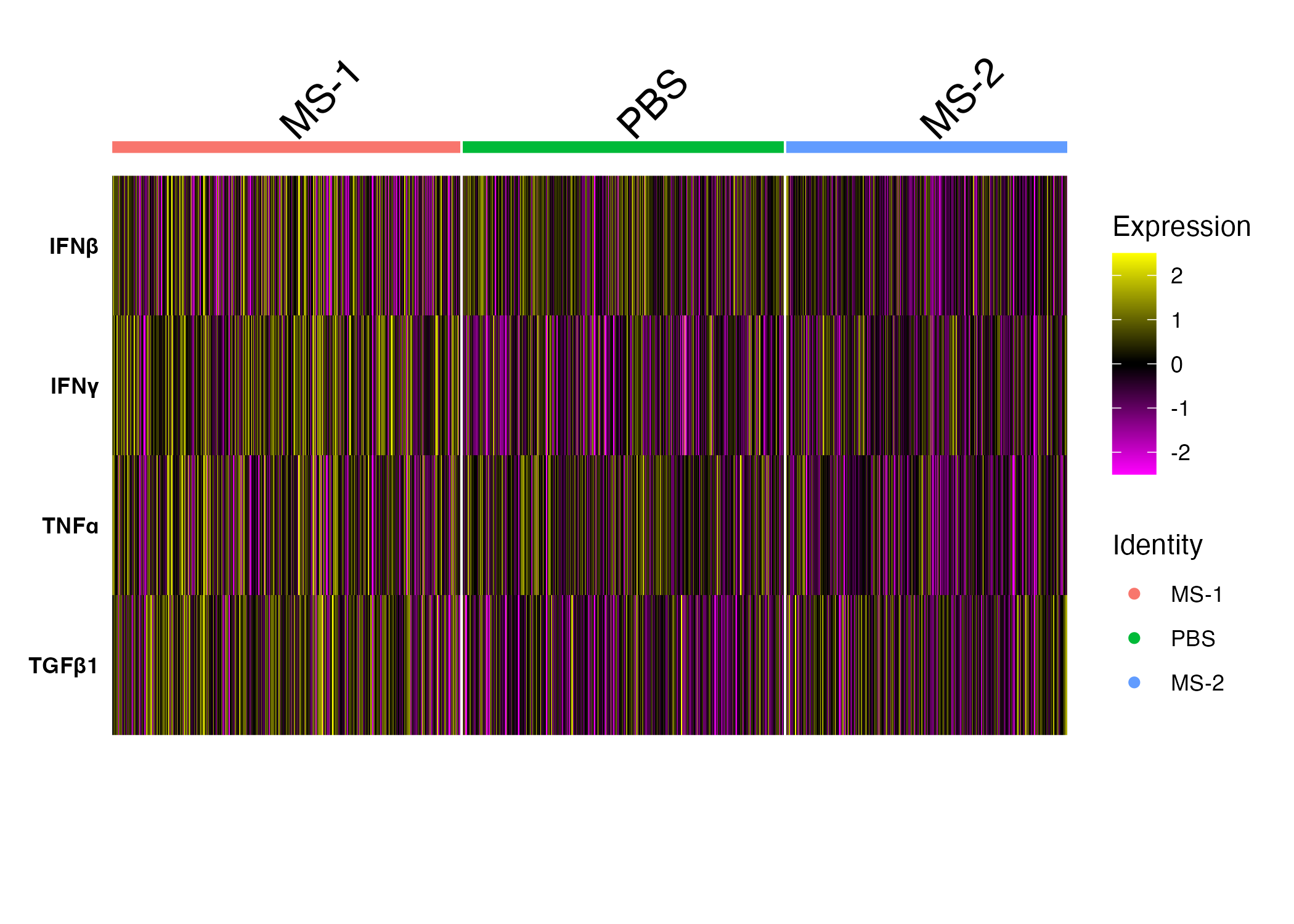

Supplement: S40 Fig — (TIFF) [file pcbi.1013475.s040.tiff]
