# Supplementary material for: Mouse-Specific Single cell cytokine activity prediction and Estimation (MouSSE)
Source: PLoS Comput Biol. 2025 Sep 19;21(9):e1013475. doi: 10.1371/journal.pcbi.1013475 (PMC12469201; doi:10.1371/journal.pcbi.1013475)
Supplement: Attachment — Submitted filename: MouSSE-ReviewerResponse-AJ_RF.pdf [file pcbi.1013475.s041.pdf]

Reviewers' comments:

We appreciate the thoughtful feedback from the reviewers on our manuscript.

We have made seven major updates to the manuscript in response to the reviewer feedback:

1. We have extended the comparison of the MouSSE method against MouSSE positive weighted (mousse.pos), MouSSE negative weighted (mousse.neg), normalized ligand score (naïve), normalized receptor score (receptor), ligand-receptor product score (product), NICHES (niches), Seurat Perturbation Scores (Seurat), PROGENy (progeny), IREA Positive-Weighted Scores (irea.pos) and IREA Positive and Negative-Weighted Scores (irea.mousse) methods for the PR-AUC, balanced accuracy, sensitivity, detection rate, F1 score, detection prevalence, negative predicted value, precision, specificity and prevalence metrics in addition to the AUC-ROC. For each metric, we quantify the proportion of cytokines with the highest metric-specific score for the method under examination. This comparison now extends the validation of MouSSE and comparative frameworks on 11 metrics. We report our results in the section titled 'Assessment using classification performance metrics.' [Reviewer 1 and Reviewer 2 and Reviewer 3]
2. We provide the associated mathematical definitions for each of the AUC-ROC, PR-AUC, balanced accuracy, sensitivity, detection rate, F1 score, detection prevalence, negative predicted value, precision, specificity and prevalence in a Section titled 'Performance metrics definition' in the Supplement. [Reviewer 1 and Reviewer 2 and Reviewer 3]
3. We note that we had initially implemented reverse cross-validation to challenge the model's ability to learn from a limited training subset and evaluate whether the derived gene sets could effectively generalize to a substantially larger test set, thereby testing robustness under data-scarce conditions. We have now implemented k/5-fold cross validation (i.e., training with k-1 folds and validating on one-fold) in our manuscript. We report that our test data consists of approximately 77,033 samples and our training data consists of approximately 308,141 samples. [Reviewer 1]
4. We have now added sensitivity analysis of MouSSE to gene set size, varying from 20, 60, 100 and 200 genes. To further analyze differences in gene set size against the average log2FC threshold, we show the distribution of the minimum absolute average log2FC threshold for each of the 86 cytokines across all gene set size ranges. We report these results in the section titled 'Sensitivity analysis of MouSSE to gene set size' of our revised manuscript. [Reviewer 2]
5. We have now corrected the NaN propagation issue in the *mousse()* function of our previous iteration of the MouSSE R package. We provide further details about the correction mechanism in the response associated with the reviewer comment. In addition, we now provide detailed instructions on our Github page [https://github.com/azkajavaid/MousseR-package] regarding the installation of the MouSSE R package and provide details for implementing the functions within the package to generate single-cell level cytokine activity estimates. [Reviewer 1 and Reviewer 3]
6. We have now extended the validation of our MouSSE method on COVID19 data generated using multiple replicates for each of the three mild and six severe COVID19 patients and three control patients. We have applied a quality control pipeline, like the one implemented by the original authors (Liao et al.), to remove low-quality samples. Our revised dataset now consists of 63,734 samples and 2,000 features. We report our updated findings in the section titled 'Application of MouSSE to COVID19 scRNA-seq data' of our revised manuscript. [Reviewer 3]
7. We now provide the PR-AUC comparison values between every pair of cytokines for the 86 evaluated cytokines for the model trained to distinguish one cytokine against every other cytokine. This additional analysis enables examination of our model's ability to correctly predict the cytokine activity signature for a cytokine under examination relative to every other cytokine. We report these results in the section titled 'Assessment using the Precision-Recall Area Under the Curve (PR-AUC)' of our revised manuscript. [Reviewer 2 and Reviewer 3]

Responses to each of the reviewer's comments are outlined in the sections below (reviewer comments are in normal font and our response follow in red font).

Reviewer's Responses to Questions

Reviewer #1: Javaid et al developed a method called MouSSE to predict mouse cytokine activities in single cell data or spatial transcriptome data. The method is based on large-scale perturbation scRNA-seq data for 86 murine cytokines. The method is evaluated by comparing with 9 other strategies. The method is applied to COVID single cell data and lymph node spatial transcriptomic data.
While it's appreciated that the results are in details and the structure of the manuscript is clear, there are some flaws in the manuscript affecting full understanding of the story, especially about the methodology and the application range of the method. This manuscript could be further considered if the following comments are addressed.

1. References are not organized properly.
(1) The indexes in the main text are in a messy order. For example, the first reference is No.3 and the second one is No.20.

Thank you for the comment. We had previously sorted references by year, name and title. We have now adjusted for this and sorted the references by appearance in the manuscript.

(2) Many sentences in the introduction section needs proper references while they are missing. Please check every sentence in the introduction section and add necessary references.

Thank you for the comment. We have thoroughly checked every sentence of the introduction section and accordingly added additional references.

(3) Also, the reference format in the reference section needs to be corrected.

Thank you for the comment. We have now correctly used the Vancouver citation format in the reference section.

2. (Page 4, section 2.5) Usually k-fold cross validation means training with k-1 folds and validating on 1 fold. Please explain why the setting in this manuscript is the opposite.

We thank the reviewer for the correction. We realized that we had previously implemented reverse cross-validation as opposed to k-fold cross-validation traditionally implying training with k-1 folds and validating on one-fold. We initially implemented reverse cross-validation to challenge the model's ability to learn from a limited training subset and evaluate whether the derived gene sets could effectively generalize to a substantially larger test set, thereby testing robustness under data-scarce conditions. We have now implemented k/5-fold cross validation (i.e., training with k-1 folds and validating on one-fold) in our manuscript. We report that our test data consists of approximately 77,033 samples and our training data consists of approximately 308,141 samples. We have adjusted all results for MouSSE and all other comparative methods accordingly.

3. (Page 7, figure 2)
(1) The ability to "correctly predict the cytokine label corresponding to each sample" is different from "correctly evaluate the cytokine activity (score)". Being able to do the former could not prove the ability to do the latter. The authors are supposed to use a strategy that could directly compare the evaluation of relative cytokine activity.

We thank the reviewer for the comment. One challenge with correctly evaluating the cytokine activity scores is that extensively documented ground truth data that provides the individual, mutually exclusive continuous activity scores for different cytokines does not currently exist. To be able to exactly evaluate the cytokine activity estimates generated by MouSSE and comparative methods would entail conducting perturbation experiments where each cytokine is individually injected and tracked (i.e., labelled with a fluorescent antibody) to ensure documentation of its continuous cytokine activity before it potentially transforms into a different cytokine downstream. We are not currently aware of a database of experiment-based cytokine activity scores that would allow us to completely distinguish the activity of one cytokine against every other cytokine. We note this as a potential limitation of our manuscript and an inherent challenge in validating cytokine activity estimates in the absence of definitive ground truth data, although we believe the available indirect evaluations still provide valuable insight into the relative performance of MouSSE. We note this limitation in our manuscript in a subsection titled 'Limitations.'

(2) ROC or AUC is used to evaluate classification problems. Here if the prediction output is a score, it's not proper to use AUC to evaluate to evaluate the performance. If the prediction is a multi-class problem, it's better to notify if the AUC is from "1 vs rest" or between two specific classes.

Thank you for the comment. We note that we used the AUC-ROC since it is used to evaluate classifiers that output continuous probabilities at different classification thresholds. AUC-ROC provides a robust evaluation of our model since instead of using a single threshold for the classification task, we are constructing a ROC curve to evaluate the drawback between the true positive rate (TPR) and the false positive rate (FPR) at various threshold levels. In this case, we are using a one-vs-rest AUC-ROC to evaluate the classification performance of correctly distinguishing the cytokine activity signature for one cytokine against all other

cytokines. We have clarified this implementation in the ‘Performance metrics’ section of our revised manuscript. To further expand on the reviewer comment, we supplement the AUC-ROC with additional metrics, including PR-AUC, sensitivity, specificity, negative predicted value, precision, F1 score, prevalence, detection rate, detection prevalence, and balanced accuracy. These metrics allow us to further assess the robustness of our models. We report the proportion of cytokines with the highest AUC-ROC, PR-AUC, balanced accuracy, sensitivity, detection rate, F1 score, detection prevalence, negative predicted value, precision, specificity and prevalence across each of the 10 methods as computed from cross-validated mouse lymph node target scRNA-seq data in Figure 4 of our revised manuscript in the section titled ‘Assessment using classification performance metrics.’ In addition, we provide the PR-AUC comparisons between every pair of cytokines for the 86 evaluated cytokines for the model trained to distinguish one cytokine against every other cytokine. This additional analysis enables examination of our model's ability to correctly predict the correct cytokine activity signature for a cytokine under examination relative to every other cytokine. We report these results in the section titled ‘Assessment using the Precision-Recall Area Under the Curve (PR-AUC)’ in our revised manuscript.

(3) Here it’s better to directly show some scores or prediction output for each cytokine by these methods to compare relative ability of different methods. It’s not informative to show just a fraction of highest AUC counts. Having better performance than other methods may not mean a good method, especially when the evaluation approach is not suitable for other methods. The readers are supposed to be informed directly at least how well this method itself could do the prediction by quantification results.

Thank you for the comment. We now provide the raw average AUC-ROC scores for all 86 cytokines quantified using the MouSSE and comparative methods as computed on the cross validated mouse lymph node scRNA-seq data in Figure 3 of our revised manuscript. In addition, we have expanded the evaluation of our model to additional metrics, including PR-AUC, sensitivity, specificity, negative predicted value, precision, F1 score, prevalence, detection rate, detection prevalence, and balanced accuracy. We provide the scores for each metric as computed for all 86 cytokines in Figures S1-S10 of our revised manuscript.

4. (Page8, figure 3)
(1) There is a row named “PBS” in the heatmap, it’s confused if this refers to a cytokine or the buffer. It it’s the buffer for negative control, then it’s unclear why there is a row showing the result for the buffer.

Thank you for your comment. We have now removed the row named “PBS” from the heatmaps and from the aggregated barplot-based results. We report the updated heatmap in Figure 3 of our revised manuscript.

(2) It looks like some AUC value is around 0.5 or even lower than 0.5, which means the evaluation is no good than a random guess. Just like question 5, it’s not meaningful to rank No.1 in the prediction score, if all methods have low AUC scores. It’s meaningful only when the method could have a good performance on the prediction.

Thank you for your comment. We report that all AUC-ROC values are at least 0.56 for activity estimates generated using our MouSSE method with a gene set size of 60 genes. We have now also included the raw AUC-ROC values in the heatmap for an easier comparison in Figure 3 of our revised manuscript.

(3) Some methods only have AUC for a small fraction of cytokines, in this case, it’s meaningless to declare that MouSSE have higher AUC than them. For example, the authors state that “Figure 2b shows that approximately 90% of cytokines (n=78) have the highest AUC score when estimated using the MouSSE method as compared to the IREA and Seurat-based strategies”, while the Seurat based method only have 4 valid AUC scores in the heatmap.

Thank you for your suggestion. We recognize that comparing AUC-ROC scores may be less meaningful when a method only produces valid AUC-ROC values for a small fraction of cytokines. However, our intent is to provide a comprehensive comparison across all cytokines for which AUC-ROC values can be computed. For transparency, we include all available data in the heatmap, even when the number of valid AUC-ROC scores for some methods is limited. We have noted this potential limitation in the section titled ‘Limitations’ in our revised manuscript.

5. (Section 4.2)
(1) Applying the method on human COVID data and show that the trend for some specific cytokines/pathways meet previous reports in figure 4-7 is not a good way to validate that the method could be applied on human. A rigorous validation requires comparison of prediction of all cytokines with previous human data quantitatively and demonstration that the method out-performs all previous methods working on human data like section 4.1.

Thank you for your suggestion. We agree that rigorous validation on human data requires systematic, quantitative comparison against prior studies and existing methods. In our previous work, we developed a method specifically for human cytokine activity estimation named SCAPE (Single cell transcriptomics-level Cytokine Activity Prediction and Estimation), which we evaluated extensively using perturbed simulated human scRNA-seq data [https://doi.org/10.1101/2023.10.17.562739]. SCAPE was benchmarked against several cytokine activity estimation methods and gene set testing approaches, showing strong performance but is currently limited to 41 cytokines. In comparison, MouSSE has been extensively validated on mouse scRNA-seq data. Our application of MouSSE to human COVID19 data was largely exploratory, intended to demonstrate its broader coverage (i.e., for

up to 86 cytokines) rather than to serve as a comprehensive human-specific validation. We agree that meaningful cross-species application will require careful attention to gene and cytokine nomenclature, and we plan to incorporate robust gene ortholog mapping strategies in future versions. We have added this point to the 'Future Directions' section.

(2) The authors are also supposed to explain in more details how the method works on non-mouse species, when the training is done on mouse data. For example, the nomenclature of cytokine names could be different in different species and it's unclear how the map from mouse gene names to other species is done. Also, it's unclear whether this method could be directly used on more species beyond mouse and human, like other mammals or non-mammals.

Thank you for your suggestion. In this manuscript, we focus exclusively on mouse data and do not perform an application of the MouSSE method on non-mouse species, as this is beyond the scope of our current work. However, we acknowledge that extending the method to other species would require careful handling of differences in gene and cytokine nomenclature. In future work, we plan to incorporate support for cross-species applications, including robust gene ortholog mapping strategies. We have noted this feedback in the section titled 'Future Directions.'

6. It's appreciated to see the authors include the download approach in GitHub. However, the authors are suggested to write the step-by-step protocol on how to run MouSSE directly in README file on GitHub. It's not easy for researcher not familiar with R to find out how to run this package.

Thank you for the comment. We have now added additional instructions on our Github page that detail the installation of the MouSSE R package and provide further details for implementing the functions within the package to generate single-cell level cytokine activity estimates. We now directly interface our vignette and provide detailed guidance on applying MouSSE on sample publicly available dataset in the README file on Github [https://github.com/azkajavaid/MousseR-package].

Reviewer #2: Strengths:

Novelty: MouSSE addresses key limitations of the authors' prior method (SCAPE) by expanding cytokine coverage (86 vs. 41), using murine-specific scRNA-seq perturbation data, and incorporating bidirectional gene weights.

Technical Rigor: The stratified cross-validation on a large-scale dataset (385k cells) and comparisons against nine alternative methods (e.g., NICHES, PROGENy) are comprehensive.

Biological Relevance: Applications to COVID-19 and ST data yield results consistent with literature (e.g., IFN-I upregulation in severe COVID-19), showcasing practical utility.

Reproducibility: Code and datasets are publicly accessible, and the method is implemented as an R package.

Weaknesses and Concerns:

Parameter Justification: The selection of the top 60 genes per cytokine (based on log2 fold-change) is arbitrary. Sensitivity analyses or rationale for this threshold (e.g., stability across gene set sizes) are absent.

Thank you for your suggestion. We have now added sensitivity analysis to gene set size, varying from 20, 60, 100 and 200 genes, for our MouSSE method. We describe these results in section titled 'Sensitivity analysis of MouSSE to gene set size' of our revised manuscript. We report the proportion of cytokine activity estimates that have each of the highest AUC-ROC, PR-AUC, balanced accuracy, F1 score, negative predicted value and precision when scored using the MouSSE method as compared to the rest of the methods for gene sets including the top 20, 60, 100 and 200 genes by absolute average log2FC threshold in Figures S22, S23, S24 and S25, respectively. To further analyze differences in gene set size against the average log2FC threshold, we also show the distribution of the minimum absolute average log2FC threshold for each of the 86 cytokines across all gene set size ranges. We report this result in Figure 5 of our revised manuscript.

Biological Validation: The study relies solely on computational metrics (AUC, correlation with Hallmark pathways). Independent experimental validation (e.g., protein-level cytokine measurements) would strengthen claims.

Thank you for your comment. One challenge with correctly evaluating the cytokine activity scores is that extensively documented ground truth data that provides the individual, mutually exclusive continuous activity scores for different cytokines does not currently exist. To be able to exactly evaluate the cytokine activity estimates generated by MouSSE and comparative methods would entail conducting perturbation experiments where each cytokine is individually injected and tracked (i.e., labelled with a fluorescent antibody) to ensure documentation of its continuous cytokine activity before it potentially transforms into a different cytokine downstream. We are not currently aware of a database of experiment-based cytokine activity scores that would allow us to completely distinguish the activity of one cytokine against every other cytokine. We note that biological experimental validation of the MouSSE and comparative methods is out of scope for our lab, but we have noted this as a potential limitation of our manuscript and an inherent challenge in validating cytokine activity estimates in the absence of definitive ground truth data, although we believe the available indirect evaluations still provide valuable insight into the relative performance of MouSSE. We detail this limitation in our manuscript in a subsection titled 'Limitations.'

To further expand on the reviewer comment, we supplement the AUC-ROC with additional metrics, including sensitivity, specificity, negative predicted value, precision, F1 score, prevalence, detection rate, detection prevalence, and balanced accuracy. These metrics allow us to further assess the robustness of our models. We report the proportion of cytokines with the highest AUC-ROC, PR-AUC, balanced accuracy, sensitivity, detection rate, F1 score, detection prevalence, negative predicted value, precision, specificity and prevalence across each of the 10 methods as computed from cross-validated mouse lymph node target scRNA-seq data in Figure 4 of our revised manuscript. In addition, we provide the PR-AUC comparisons between every pair of cytokines for the 86 evaluated cytokines for the model trained to distinguish one cytokine against every other cytokine. This additional analysis enables examination of our model's ability to correctly predict the correct cytokine activity signature for a cytokine under examination relative to every other cytokine. We report these results in the section titled 'Assessment using the Precision-Recall Area Under the Curve (PR-AUC)' in our revised manuscript.

Overfitting Risks: Gene sets are derived from the Immune Dictionary, which is also used for cross-validation. Testing on entirely independent datasets (beyond Liao and Lopez et al.) would better assess generalizability.

Thank you for your suggestion. We recognize the importance of evaluating generalizability beyond datasets used in training or cross-validation. To further assess the robustness of MouSSE, we evaluate its performance using 10 additional metrics, including the F1-score, which is particularly informative in the case of imbalanced datasets. While these additional metrics provide a more comprehensive assessment of model performance, we recognize that validation on an additional dataset would further strengthen confidence in the method's generalizability. We are currently exploring the feasibility of such analyses and would be open to incorporating additional datasets, potentially in collaboration with the reviewer or in future work.

Cell-Type Heterogeneity: The cell-type-agnostic approach may obscure cell-specific signaling dynamics. The discussion acknowledges this limitation but does not quantify its impact on results.

Thank you for your suggestion. Unfortunately, we could not incorporate cell-type-specific signaling dynamics since we were not able to obtain data relevant to cell type specific signatures from the authors of the Immune Dictionary. We plan to explore the cell type-specific models in future work (either using cell type annotations provided by the Immune Dictionary authors or independently generated cell type labels). We have also noted this in the 'Future Directions' section of our revised manuscript.

Methodological Clarity: The modified VAM's gamma distribution fitting and its contribution to scoring are under-explained. A pseudocode or schematic would improve transparency

Thank you for the feedback. We have now provided further detail about the implementation of the modified Mahalanobis distance in VAM as compared to the standard Mahalanobis distance in the subsection titled 'Gene set scoring.' Specifically, we note that the modified Mahalanobis distance implementation in VAM has two important differences from the standard Mahalanobis distance implementation. First, while the standard Mahalanobis distance leverages the full sample covariance matrix for estimation, the modified Mahalanobis distance only accounts for the technical variance of each gene, thereby ignoring covariances. Practically, this implies that VAM only discounts deviations in directions of large estimated technical variance while preserving deviations in directions of large biological variance (i.e., covariance). Second, while the standard Mahalanobis distance calculates distances from the multivariate mean, the modified Mahalanobis distance calculates distances from the origin, thereby resulting in more biologically plausible distance measure for scRNA-seq data.

How does MouSSE handle cytokines with overlapping gene signatures (e.g., IL-4/IL-13)? Does the differential expression analysis against all other cytokines sufficiently mitigate redundancy?

We appreciate the reviewer comment. To further investigate overlapping gene signatures, we now provide the PR-AUC comparisons between every pair of cytokines for the 86 evaluated cytokines for the model trained to distinguish one cytokine against every other cytokine. This additional analysis enables examination of our model's ability to correctly predict or distinguish the correct cytokine activity signature associated with a cytokine under examination relative to every other cytokine. We report these results in the section titled 'Assessment using the Precision-Recall Area Under the Curve (PR-AUC)' in our revised manuscript.

Why does the positive-weighted of MouSSE perform comparably to the full method in ST data but not in COVID-19 scRNA-seq? Is this due to dataset sparsity or biological context?

We thank the reviewer for the insightful question. One possible explanation for the comparable performance of the positive-weighted MouSSE with the full MouSSE in spatial transcriptomics (ST) data, but not in the COVID19 scRNA-seq data, relates to differences in data sparsity and structure between these modalities. ST data typically aggregates gene expression across multiple cells within spatial spots, resulting in lower sparsity and smoother expression profiles. These smoothed expression profiles can enhance the signal of positively weighted immune gene sets, allowing the positive-only model to perform comparably to the full version of MouSSE. In contrast, scRNA-seq is highly sparse and heterogeneous, capturing fine-grained cellular differences. For scRNA-seq data, therefore, incorporating both positively and negatively weighted genes helps distinguish more nuanced immune programs, which are better captured by the full MouSSE model.

Include sensitivity analyses for key parameters (e.g., gene set size, fold-change thresholds).

Thank you for your suggestion. We have now added sensitivity analysis to gene set size, ranging from 20, 60, 100 and 200 genes, for our MouSSE method. We describe these results in section titled 'Sensitivity analysis of MouSSE to gene set size' of our revised manuscript. We report the proportion of cytokine activity estimates that have each of the highest AUC-ROC, PR-AUC, balanced accuracy, F1 score, negative predicted value and precision when scored using the MouSSE method as compared to the rest of the methods for gene sets including the top 20, 60, 100 and 200 genes by absolute average log2FC threshold in Figures S22, S23, S24 and S25, respectively. To further analyze differences in gene set size against the average log2FC threshold, we also show the distribution of the minimum absolute average log2FC threshold for each of the 86 cytokines across all gene set size ranges. We report this result in Figure 5 of our revised manuscript.

Discuss limitations of AUC (e.g., class imbalance in cytokine labels) and supplement with precision-recall curves if applicable.

We appreciate the reviewer suggestion. To further expand on the reviewer comment, we supplement the AUC-ROC with additional metrics, including sensitivity, specificity, negative predicted value, precision, F1 score, prevalence, detection rate, detection prevalence, and balanced accuracy. These metrics allow us to further assess the robustness of our models, especially for class imbalanced data. We report the proportion of cytokines with the highest AUC-ROC, PR-AUC, balanced accuracy, sensitivity, detection rate, F1 score, detection prevalence, negative predicted value, precision, specificity and prevalence across each of the 10 methods as computed from cross-validated mouse lymph node target scRNA-seq data in Figure 4 of our revised manuscript and in the section titled 'Assessment using classification performance metrics.' In addition, we provide the PR-AUC

comparisons between every pair of cytokines for the 86 evaluated cytokines for the model trained to distinguish one cytokine against every other cytokine. This additional analysis enables examination of our model's ability to correctly predict the correct cytokine activity signature for a cytokine under examination relative to every other cytokine. We report these results in the section titled ‘Assessment using the Precision-Recall Area Under the Curve (PR-AUC)’ in our revised manuscript. Lastly, we further discuss the utility of using metrics like F1 score, balanced accuracy and PR-AUC, especially for imbalanced dataset, in our revised manuscript.

Expand the biological interpretation: How might MouSSE’s predictions inform therapeutic strategies (e.g., targeting IFN-I in severe)?

Thank you for your comment. Our MouSSE method can be leveraged in clinical settings to generate single-cell level cytokine activity estimates from patient transcriptomics data. These estimates can inform therapeutic strategies by identifying dominant cytokine-driven pathways in a patient's immune response. For example, in the context of severe COVID19, if MouSSE identifies elevated IFN-I activity in specific immune cell subsets, clinicians might consider targeting IFN-I signaling with JAK inhibitors or other pathway-specific modulators. More broadly, this patient-level cytokine profiling could be used to stratify individuals by inflammatory risk or cytokine response profiles, enabling personalized immunomodulatory treatment plans. We note this application of MouSSE in the ‘Discussion’ section of our revised manuscript.

Reviewer #3: MouSSE is a well-motivated attempt to quantify cytokine activity in scRNA-seq data by combining murine stimulation signatures with a variance-adjusted Mahalanobis (VAM) score. Benchmarking against nine competing tools and two external datasets shows promising average AUC gains. The idea is sound and potentially valuable, but the current manuscript and code require substantial revisions before the work can be considered robust and reproducible.

Major issues that must be addressed:

1. Incomplete capture of canonical biology (IL-6 in severe COVID-19): IL-6 is universally recognized as the hallmark cytokine of severe COVID-19, its blood concentrations correlate best with respiratory failure and mortality, and antagonists such as tocilizumab are among the few immunomodulators with proven clinical benefit. Any tool that claims to quantify cytokine activity in single-cell data should therefore detect a clear IL-6 signal in broncho-alveolar lavage from severe cases. Your current results do not highlight IL-6, which calls into question the biological validity of the murine signatures, the weighting scheme, or the averaging strategy. Resolving this discrepancy is essential before the approach can be considered reliable.

- Please verify that the IL-6 module is active, consider whether cell-type-restricted expression, single-cell dropout (secreted cytokine) or the murine training signatures could mask the signal, and explain or correct this discrepancy.

Thank you for your comment. In light of reviewer feedback, we reviewed the COVID19 data and noted that Liao et al. applied a specific quality control pipeline to the COVID19 dataset when combining data from the six severe and three mild COVID19 patients and three control patients. Whereas we were previously considering data from a patient from each of the severe and mild COVID19 and control categories, we have now updated our data processing methodology to include data from all six severe and three mild COVID19 patients and three control patients using the recommended quality control procedure. Following quality control, we merged data across replicates and integrated across disease severity. We then applied MouSSE and comparative methods on this dataset consisting of 63,734 samples and 2,000 features. We report the top 15 cytokine markers by average log2FC that are differentially expressed based on COVID19 severity condition in Figure 6 of our revised manuscript. We find that these updated results show a much specific upregulation signature for IL6 for severe COVID19 as compared to control and mild COVID19 for activity estimates generated using MouSSE. In addition, we observe stratification of differentially expressed markers by different patient-specific samples within the severe COVID19 condition. For example, differentially expressed markers specific to the severe-c149 and severe-c152 patients show some overlap with differentially expressed markers identified for the mild COVID19 and control patients. On further inspection, we find that while patients c149 and c152 both had severe COVID19, they were relatively younger than their other severe counterparts. Patient c149 was 57 years old, for example, while patient c152 was 46 years old. In comparison, most other severe COVID19 patients were in their 60s (i.e., severe-c143, severe-c145, severe-c146 and severe-c148 patients were 62, 66, 63 and 65 years old, respectively). We find support for our assertion by Liao et al. as they report that while patient c152 was previously in critical condition, they later improved.

- Figure 4b suggests IL-6 is higher in the severe group, yet IL-6 is not discussed. What are the $\log_2$ fold-change and FDR-adjusted P values for IL-6 under both the full MouSSE score and the positive-only variant?

Thank you for your comment. We now extensively discuss the upregulation of IL6 in the severe group for activity estimates generated using the MouSSE and comparative methods using our updated quality control methodology. Please see comments below for comparison of IL6 expression for the full MouSSE and the positive-only variant.

- Are rows in the heatmaps filtered by statistical significance before plotting? Please specify the exact thresholds so readers can interpret colour gradients relative to inclusion in the narrative.

Thank you for your comment. Following estimation, we scale the generated cell signaling estimates and visualize the top 15 cytokine markers by average log2FC that are differentially expressed based on COVID19 severity condition (i.e., control, mild and severe).

- The positive-only score appears to recover a canonical severe-COVID-19 cytokine that the full score attenuates, yet you conclude the signed-weight model is superior overall. How do you reconcile this single-case discrepancy with the aggregate AUC advantage you report?

We appreciate the reviewer observation. We report that while the results generated by MouSSE support the expected upregulation of canonical markers of COVID19 by disease severity and the expected stratification within COVID19 severity categories, the results generated by comparative methods do not support a similar pattern of findings. For example, we find that while interferon-specific cytokines like IFNα1 and IFNγ are upregulated in patients with severe COVID19 for activity estimates generated using the mousse.pos (see Figure S26) and mousse.neg (see Figure S27) methods, there is not necessarily a strict, clustered separation between differentially expressed markers by disease severity. Similarly, there is not a strict upregulation of markers specific to severe COVID19 (i.e., IFNα1, IFNβ, IFNγ and IL6) relative to control and mild COVID19 for differentially expressed markers generated using the cytokine-specific estimates produced by the irea.pos method (see Figure S28). We do find however that the implementation of our gene set scoring strategy with the IREA-specific gene sets (i.e., irea.mousse) considerably improves the performance of the IREA method (see Figure S29). More specifically, we find that interferon-specific cytokines like IFNα1 and IFNβ are correctly upregulated in severe COVID19 as compared to control and mild COVID19. Additionally, these markers are not strictly upregulated in severe c149 and severe c152 patients, which is a result that is

supported by our MouSSE method. We also find that IL6 is not necessarily upregulated in severe COVID19 as compared to control and mild COVID19 for cytokine activity estimates generated using the irea.mousse method. In this regard, MouSSE is relatively better and more specific than the cyokine activity estimates generated using the irea.mousse and comparative methods since MouSSE generates cytokine activity estimates that result in identification of IL6 as a distinct marker for severe COVID19. We also report that activity estimates generated using the naive (Figure S30), receptor (Figure S31), product (Figure S32), Seurat (Figure S33) and PROGENy methods (Figure S34) are relatively noisy and less informative than other gene set-specific methods, thus failing to fully account for signaling variations by disease severity.

- Could negative-weight genes derived from mouse lymph-node stimulation invert or dampen the IL-6 signal in human BALF? Would re-deriving the IL-6 signature from human in-vitro data mitigate this issue?

Thank you for your comment. To evaluate the potential impact of negative-weight genes on IL6 signal interpretation, we computed cytokine activity estimates using the full MouSSE method (which incorporates both positive and negative weights), a positive-only variant, and a negative-only variant. Our results indicate that the full MouSSE method most effectively captures the expected up-regulation of COVID19-associated signatures in relation to disease severity. These findings suggest that the inclusion of both gene sets enhances biological resolution rather than inverting the signal. We report the differential expression markers generated for our MouSSE method in Figure 6, for the positive-only variant (mousse.pos) in Figure S26 and for the negative-only variant (mousse.neg) in Figure S27 of our revised manuscript. All supporting analyses are provided in the Supplement. We agree that deriving IL6 signatures directly from human in vitro datasets could offer additional insights, and we consider this a valuable direction for future work to ensure cross-species robustness.

- Why is IL-6 not included in the Fig6 analysis?

Thank you for your observation. We have now generated updated figures that include IL6. We report the top 15 cytokine markers by average log2FC that are differentially expressed based on COVID19 severity condition for activity estimates generated using the MouSSE method in Figure 6 of our revised manuscript.

2. Cross-validation strategy and performance evaluation:
- Current five-fold splits operate at the cell level. Splitting by stimulation replicate or donor would give a fairer estimate of generalizability. Provide results for such experiment-level hold-outs. Add an independent mouse dataset from a separate laboratory.

Thank you for your suggestion. We recognize the importance of evaluating generalizability beyond datasets used in training or cross-validation. We note that we have performed stratified cross-validation, which ensures that the class distributions for each fold of the data are approximately balanced and proportional to the class distributions in the full data set. Stratified cross-validation is therefore especially beneficial for imbalanced data. However, we recognize the reviewer comment that five-fold-validation based on stimulation replicate-level split may further improve generalizability of our MouSSE framework and are happy to accommodate reviewer feedback in future version of the manuscript. We have also added a reference to this analysis in the 'Future Directions' section of our revised manuscript.

- Provide additional metrics beyond AUC, such as report PR-AUC, Spearman correlation with cytokine protein or phospho-STAT measurements, and computing resources (runtime and memory differences arise between MouSSE and the nine comparators).

Thank you for your suggestion. To further expand on the reviewer comment, we supplement the AUC-ROC with additional metrics, including sensitivity, specificity, negative predicted value, precision, F1 score, prevalence, detection rate, detection prevalence, and balanced accuracy. These metrics allow us to further assess the robustness of our models. We report the proportion of cytokines with the highest AUC-ROC, PR-AUC, balanced accuracy, sensitivity, detection rate, F1 score, detection prevalence, negative predicted value, precision, specificity and prevalence across each of the 10 methods as computed from cross-validated mouse lymph node target scRNA-seq data in Figure 4 of our revised manuscript in the section titled 'Assessment using classification performance metrics.' In addition, we provide the PR-AUC comparisons between every pair of cytokines for the 86 evaluated cytokines for the model trained to distinguish one cytokine against every other cytokine. This additional analysis enables examination of our model's ability to correctly predict the correct cytokine activity signature for a cytokine under examination relative to every other cytokine. We report these results in the section titled 'Assessment using the Precision-Recall Area Under the Curve (PR-AUC)' in our revised manuscript. We further provide the runtime complexity results in the section titled 'Sensitivity analysis of MouSSE to gene set size.'

3. NaN propagation in the public vignette: The PBMC3k example triggers NaNs produced warnings from sqrt(diag()) inside VAM. Diagnose the cause (are these zero variance genes), document how vamForCollection handles NaNs, and implement or describe pre-filters that prevent silent score loss.

Thank you for your comment. We realized that the *mousse()* function previously released in the Github repository was setting the positive weights to the gene indices, which have a lot of variation for each set, as opposed to the actual weights. We report that this does not impact the results presented in the manuscript and only impacts the vignette and the associated functions released on the Github repo. We have now released an updated version of the MousseR package and report that this update does not

trigger the NaN warning previously propagated by the *vamForCollection()* function inside VAM. We have also added additional instructions on our Github page [https://github.com/azkajavaid/MousseR-package] that detail the installation of the MouSSE R package and provide details for implementing the functions within the package to generate single-cell level cytokine activity estimates.

4. Manuscript formatting and incorrect statements:
- No line numbers

Thank you for your comment. We have now added line numbering.

- Numerous missing references (Introduction starts at ref 3, then jumps to 20).

Thank you for your comment. We had previously sorted references by year, name and title. We have now adjusted for this and sorted the references by appearance in the manuscript. We have also thoroughly checked every sentence of the introduction section and accordingly added additional references.

- Introduction oversimplifies pro-inflammatory cytokines by omitting their pathogen-clearance role

Thank you for your suggestion. We have now added additional text in the Introduction section, clarifying on the importance of both pro- and anti-inflammatory cytokines for pathogen-clearance and regulating the immune response.

- IFN-γ is not type I IFN

Thank you for your suggestion. We have corrected for this in our updated version of the manuscript.

5. Data and code availability: Source datasets are public and the MouSSE R package is on GitHub, but the current vignette's NaN warnings undermine trust. Resolve the warnings, document causes and mitigation and verify that all figure-generation scripts reproduce the manuscript results without manual tweaks. Consider uploading full pipelines, pre-computed gene sets to Zenodo to obtain doi-backed reference to ensure reproducibility.

Thank you for your comment. We have now resolved the NaN warning. We also have the implemented gene sets provided within our MouSSE R package accessible with the *genesetImmune()* function on Github [https://github.com/azkajavaid/MousseR-package].
